# Interlimb Transfer of Reach Adaptation Does Not Require an Intact Corpus Callosum: Evidence from Patients with Callosal Lesions and Agenesis

**DOI:** 10.1523/ENEURO.0190-20.2021

**Published:** 2021-07-26

**Authors:** Penelope A. Tilsley, Patricia Romaiguère, Eve Tramoni, Olivier Felician, Fabrice R. Sarlegna

**Affiliations:** 1 Aix Marseille Univ, CNRS, ISM, Marseille, France; 2 Aix Marseille Univ, INSERM, INS, Inst Neurosci Syst, Marseille, France; 3 APHM, CHU de la Timone, Service de Neurologie et Neuropsychologie, Marseille, France

**Keywords:** corpus callosum, intermanual transfer, motor learning, prismatic adaptation, reaching, split-brain

## Abstract

Generalization of sensorimotor adaptation across limbs, known as interlimb transfer, is a well-demonstrated phenomenon in humans, yet the underlying neural mechanisms remain unclear. Theoretical models suggest that interlimb transfer is mediated by interhemispheric transfer of information via the corpus callosum. We thus hypothesized that lesions of the corpus callosum, especially to its midbody connecting motor, supplementary motor, and premotor areas of the two cerebral hemispheres, would impair interlimb transfer of sensorimotor adaptation. To test this hypothesis, we recruited three patients: two rare stroke patients with recent, extensive callosal lesions including the midbody and one patient with complete agenesis. A prismatic adaptation paradigm involving unconstrained arm reaching movements was designed to assess interlimb transfer from the prism-exposed dominant arm (DA) to the unexposed non-dominant arm (NDA) for each participant. Baseline results showed that spatial performance of each patient did not significantly differ from controls, for both limbs. Further, each patient adapted to the prismatic perturbation, with no significant difference in error reduction compared with controls. Crucially, interlimb transfer was found in each patient. The absolute magnitude of each patient’s transfer did not significantly differ from controls. These findings show that sensorimotor adaptation can transfer across limbs despite extensive lesions or complete absence of the corpus callosum. Therefore, callosal pathways connecting homologous motor, premotor, and supplementary motor areas are not necessary for interlimb transfer of prismatic reach adaptation. Such interlimb transfer could be mediated by transcallosal splenium pathways (connecting parietal, temporal and visual areas), ipsilateral cortico-spinal pathways or subcortical structures such as the cerebellum.

## Significance Statement

Theoretical models suggest that interlimb transfer of sensorimotor adaptation is mediated by interhemispheric interactions via the corpus callosum, specifically between motor cortices. We thus hypothesized that interlimb transfer of prism adaptation in a reaching task would be impaired in patients with callosal abnormalities, especially those affecting midbody pathways connecting the motor cortices. Contrarily, we found interlimb transfer in each patient, to a level comparable to that of controls. Our findings show that callosal pathways connecting motor, premotor, and supplementary motor areas are not necessary for the interlimb transfer of prismatic reach adaptation. Alternatively, this transfer could be mediated by ipsilateral cortico-spinal pathways, subcortical structures such as the cerebellum or callosal splenium pathways connecting parietal, temporal and visual areas.

## Introduction

When we are exposed to novel properties of the body or the environment, motor behavior is optimized through trial-by-trial fine-tuning of sensorimotor neural networks, an adaptation thought to evolve through the iterative comparison of the planned and executed movements (Luauté et al., 2009; Shadmehr et al., 2010; Wolpert et al., 2011). One feature of this sensorimotor adaptation is that it is not necessarily specific to the conditions in which it was acquired, but can generalize to a different task (Morton and Bastian, 2004) or a different effector (Wang and Sainburg, 2003; Lee et al., 2010; Taylor et al., 2011; Stöckel et al., 2016; Green and Gabriel, 2018). Transfer between effectors, termed interlimb transfer, has been repeatedly evidenced in studies of upper-limb movements aiming to determine the local or global nature of the adaptive process (Harris, 1965; Dizio and Lackner, 1995; Criscimagna-Hemminger et al., 2003; Malfait and Ostry, 2004; Joiner et al., 2013; Renault et al., 2020), yet the underlying neural mechanisms remain unclear (Ruddy and Carson, 2013).

Longstanding theoretical models of the neural mechanisms of interlimb transfer highlight the key role of the corpus callosum, the largest white matter tract connecting the two cerebral hemispheres (Taylor and Heilman, 1980; Parlow and Kinsbourne, 1989, 1990). The Callosal Access Model (Taylor and Heilman, 1980) proposes that unimanual adaptation is encoded within the contralateral hemisphere and is accessible, via the corpus callosum, to the opposite hemisphere-arm system (see also Sainburg and Wang, 2002). The cross-activation model (Parlow and Kinsbourne, 1989, 1990) proposes that unimanual adaptation is encoded in the contralateral hemisphere, and copied, via the corpus callosum, to the opposite hemisphere-arm system. Lee et al. (2010) later provided neurophysiological evidence that both the contralateral and ipsilateral motor cortices are involved in both adaptation and interlimb transfer of adaptation. Perez et al. (2007a) also provided evidence that interlimb transfer of sequence learning is driven by bilateral supplementary motor areas, connected via the corpus callosum midbody (Fabri et al., 2014; Ruddy et al., 2017). Further, Perez et al. (2007b) reported that interlimb transfer was related to modulations of the transcallosal midbody pathways connecting homologous motor cortices (see also Ruddy and Carson, 2013). These studies thus suggest that the corpus callosum, and in particular its midbody segment that connects motor, supplementary motor, and premotor regions bilaterally, plays a key role in interlimb transfer.

One approach which has led to key insights into the functional role of callosal pathways has been to study neurologic individuals with corpus callosum abnormalities (Volz and Gazzaniga, 2017). Using this approach, interlimb transfer was shown to be impaired in agenesis patients and split-brain patients (de Guise et al., 1999), and multiple sclerosis patients with corpus callosum atrophy (Bonzano et al., 2011). The results of these studies are in line with the aforementioned theoretical models (Taylor and Heilman, 1980; Parlow and Kinsbourne, 1989, 1990). However, Thut et al. (1997) found interlimb transfer of proximal drawing movements in agenesis patients and a traumatic brain injury patient with corpus callosum damage. Criscimagna-Hemminger et al. (2003) also reported interlimb transfer of force-field reach adaptation in a split-brain patient, whose corpus callosum was surgically sectioned to alleviate severe epilepsy. These two studies thus cast doubt on the generalizability of the dominant theories of interlimb transfer.

The present study aimed to determine the role of the corpus callosum in the interlimb transfer of sensorimotor adaptation by assessing transfer in one patient with complete agenesis as well as two stroke patients with callosal damage. The two stroke patients presented a rare opportunity to assess the impact of recent, non-surgical callosal lesions in typically developed adults with no epilepsy. Patients and matched controls were tested on a prism adaptation paradigm involving unconstrained arm reaching movements. This paradigm, used in both fundamental and rehabilitation contexts (Harris, 1963; Martin et al., 1996a; Rossetti et al., 1998), is known to result in after-effects on the exposed arm but also on the non-exposed arm, evidencing interlimb transfer (Hamilton, 1964; Renault et al., 2020). The methodological procedure employed here was based on previous work (Harris, 1963; Dizio and Lackner, 1995; Martin et al., 1996a; Kitazawa et al., 1997; Lefumat et al., 2015) and allowed assessment of transfer for each individual (Renault et al., 2020), a critical issue when studying unique patients (Lefumat et al., 2016). Based on previous research highlighting the role of the corpus callosum in interlimb transfer, we hypothesized that patients lacking callosal connections between motor, premotor, and supplementary motor areas would show impaired interlimb transfer of sensorimotor adaptation.

## Materials and Methods

### Participants

Three patients with corpus callosum disorders (MS, MM, and AM) and 16 healthy individuals participated in the study. The number of healthy participants reflect the sample size used in similar studies (Wang and Sainburg, 2003; Morton and Bastian, 2004; Perez et al., 2007a; O’Shea et al., 2014; Lefumat et al., 2015; Leclere et al., 2019; Striemer et al., 2019; Bao et al., 2020; Fleury et al., 2020; Renault et al., 2020). Patient MS was a 50-year-old left-handed female with recently acquired lesions of the body of the corpus callosum, sparing the splenium and the genu. Patient MM was a 29-year-old right-handed male also with recently acquired lesions of the corpus callosum, sparing only the splenium. Patient AM was a 50-year-old right-handed male with complete agenesis of the corpus callosum (see Table 1; for full patient descriptions, see below). All patients and controls had normal or corrected-to-normal vision, with control participants declaring no previous or current sensorimotor or neurologic deficits. Handedness was determined using the 10-item version of the Edinburgh Handedness Inventory (Oldfield, 1971). Considering the patients’ characteristics, two control groups were recruited: group A: age = 52 ± 4 years, *n* =* *8 (five right-handed males; three left-handed females) and group B: age = 29 ± 4 years, *n *=* *8 (eight right-handed males). As developed later, the differences between the patients led us to compare each patient (instead of the group of patients) to control participants.

**Table 1:** Clinical and MRI features for each patient based on neuropsychological assessments

Patients	Proprioceptive transfer	Visual alexia	Visual anomia	Tactile anomia	Agraphia	Constructive apraxia	Ideomotor apraxia	Alien hand (diagonistic apraxia)	MRI features
MS	+	o	o	o	o	o	+ (L) [mild]	+ (R) * [weekly]	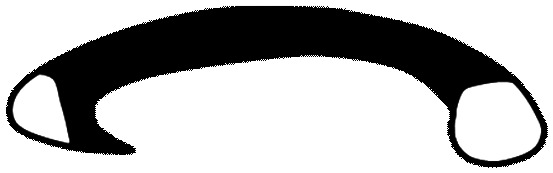
Stroke- induced lesions
MM	+	o	o	+ (L)	+ (L)	o	+ (L)	+ (L) [daily]	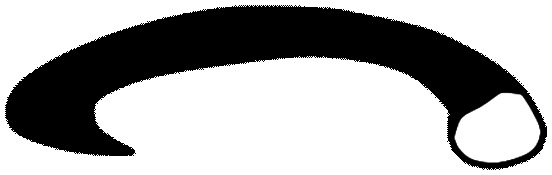
Stroke- induced lesions
AM	+	+ (R) [mild]	+ (R)	o	+ (R) [mild]	+ (R)	+ (R)	+ (R) [weekly]	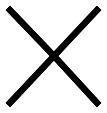
Agenesis

Columns indicate clinical features of disconnection (based on neuropsychological assessments) which were either present (+) or absent (o) in each patient, with indication of the affected arm, left (L) or right (R) when applicable. Square brackets [] are used to report when symptoms were only mild or the frequency of alien hand episodes. *Alien hand episodes for patient MS were present immediately following the stroke, but resolved six months poststroke, reoccurring only with fatigue or stress. MRI features indicate lesioned (black) and preserved (white) areas of the corpus callosum; a cross indicates complete absence of the corpus callosum from birth.

Before taking part in the experiment, participants were presented with an information sheet on the protocol, filled out the Edinburgh Handedness Inventory, and gave their written informed consent to participate. Participants could leave the experiment at any time and were free to ask questions to the experimenter; they were kept as naive as possible to the exact purpose of the study. The study was approved by the local institutional review board and performed in accordance with the standards laid out by the Declaration of Helsinki (1964).

### Patients’ profiles

**Figure 1.**
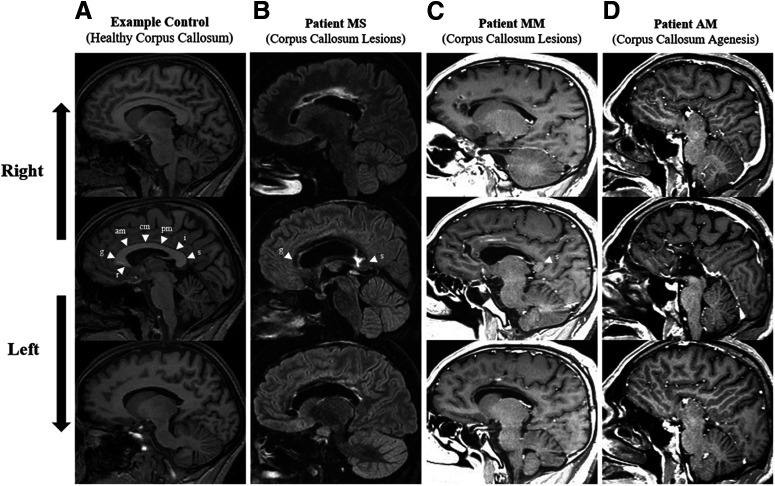
Sagittal MRI cross-section spanning from right (top row) to left (bottom row) hemisphere for (***A***) a typical control participant with complete corpus callosum (T1), (***B***) patient MS who had a brain aneurysm rupture causing lesions to the corpus callosum with only the genu (g) and splenium (s) preserved (T2-flair), (***C***) patient MM who had a stroke causing lesions to the corpus callosum with only the splenium (s) preserved (T1), and (***D***) patient AM with absent corpus callosum since birth (complete callosal agenesis; T1). Corpus callosum regions marked by white arrows are labeled on the middle row images, based on Witelson (1989), as: rostrum (r), genu (g), anterior midbody (am), central midbody (cm), posterior midbody (pm), isthmus (i), and splenium (s).

Patient MS was a left-handed female (laterality quotient: −100%), 50 years old at the time of testing (March 2017). MS had suffered from a ruptured brain aneurysm in the anterior cerebral artery 2.5 years previously at 48 years old (August 2014). This resulted in damage to the whole body of the corpus callosum, with only the anterior (genu) and posterior (splenium) regions being preserved (Fig. 1*B*), as well as hemosiderin deposits in the left and right cingulum. Patient MS thus presented a rare hemorrhagic stroke subtype (Li et al., 2015), which allowed us to study the impact of an insult to the corpus callosum in an individual with a normal development and no known neurologic disorder (e.g., no epilepsy) before the corpus callosum damage. With regards to motor function, clinical tests (see Table 1) indicated slight ideomotor apraxia in performing gestures with the left hand and impaired proprioceptive transfer between the two arms. In the months following the acute hemorrhage, she also reported recurrent conflicts between the two hands as depicted in the setting of corpus callosum injury under the terms of diagonistic dyspraxia (Akelaitis, 1945) or alien hand syndrome (Biran et al., 2006). For instance, patient MS stated that when trying to open the wardrobe with one hand to select an item of clothing, the other hand would shut it. When tested for this experiment, the patient reported that intermanual conflicts had mostly resolved, with very occasional symptoms reappearing with stress or fatigue. Neuropsychological assessments undertaken between 2015 and 2017 indicated a normal global cognitive functioning with below average attentional capacity and short-term memory.

Patient MM was a right-handed male (laterality quotient: 75%), 29 years old at the time of testing (January 2019). MM had an ischemic stroke in the territory of the bilateral anterior cerebral arteries following an intravascular thrombus in August 2018. This resulted in extensive lesions to the anterior and mid cingulate gyrus, and the rostrum, genu and body of the corpus callosum, sparing only the posterior (splenium) region (Fig. 1*C*). Clinical testing (see Table 1) showed that the patient displayed moderate motor slowing with a mild motor apraxia predominantly on the left side and occasional troubles in movement initiation. The patient also reported intermanual conflicts, with the left hand interfering with the actions performed by the right hand. For example, when opening a door with the right hand, the left hand would try to shut it. Neuropsychological assessments also revealed sustained attention and memory deficits. Patient MM thus provided another rare opportunity to study the effect of a recent lesion involving the corpus callosum in an adult with typical development.

Patient AM was a right-handed male (laterality quotient: 80%), 50 years old at the time of testing (February 2018). AM had complete congenital agenesis of the corpus callosum (Fig. 1*D*) and posterior commissure, left hippocampal sclerosis, and a history of complex partial seizures in the setting of mesial temporal lobe epilepsy. Full patient details can be found in Ridley et al. (2016), but in summary, AM endured status epilepticus in March 2012. One month later, despite full resolution of epileptic seizures, AM developed intermanual conflicts: for instance, when putting on a pair of trousers with the left hand, the right hand would pull them off (Ridley et al., 2016). Neuropsychological assessment revealed right-sided constructional apraxia, right ideomotor apraxia and right visual anomia, showing signs of interhemispheric disconnection. Global cognitive functioning was low to average. Follow-up assessments conducted in the following years indicated significant amelioration of diagonistic dyspraxia and interhemispheric disconnection features (see Table 1). Testing patient AM allowed us to explore the influence of complete absence of the corpus callosum throughout development.

### Experimental setup

**Figure 2.**
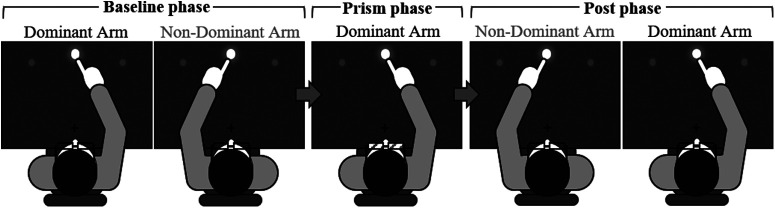
Experimental protocol, with three phases (baseline, prism, and post phase) made up of blocks of dominant (DA) or non-dominant (NDA) reaching. In the baseline phase, participants reached under normal vision from the starting point (black cross +) to one of three flashed visual targets (gray-white circles) 30 times with the DA arm, then 30 times with the NDA arm (totaling 10 trials per target per arm). In the following prism phase (exposure), participants reached 100 times (control group A, patient MS, patient AM) or 50 times (control group B, patient MM) with the DA arm toward the middle, straight-ahead visual target while wearing rightward deviating (17°) prismatic goggles. During the post phase, participants again reached under normal vision to one of three visual targets, 30 times with the NDA arm, then 30 times with the DA arm (totaling 10 trials per target per arm).

Participants were seated in front of a horizontal table positioned at waist height. The table was equipped with a raised, red start button (2 cm in diameter) located at 0° (straight-ahead) according to the body midline, directly in front of the participants chest. The start button was present at all times during the experiment. Given that the lights of the experimental room were on throughout the experiment, participants could thus both see and feel the start button position. Red light-emitting diodes (3 mm in diameter) on the table were used as visual targets (Fig. 2). Three targets were used in this study, all located 37 cm from the starting position: a middle target located at 0° (straight-ahead), a rightward target located at +20° and a leftward target located at −20° with respect to the body midline. Participants were required to wear either standard (control) goggles or altered (17° rightward deviating prismatic) goggles equipped with 30-diopter Fresnel 3M Press-on plastic lenses (3M Health Care), as used in Martin et al. (1996b). Welding goggles were used so that vision was only possible through the lenses (O’Shea et al., 2014). The use of a head restraint was avoided based on results of Hamilton (1964) showing that restraining the head precludes interlimb transfer of prism adaptation.

Infrared active markers were taped to the right and left index fingertips and their positions were sampled at 350 Hz using an optical motion tracking system (Codamotion cx1 and MiniHub, Charnwood Dynamics Ltd). The experimenter controlled the motion tracking system as well as the protocol using a customized software and a real-time acquisition system (ADwin-Pro, Jäger). An infrared camera allowed continuous real-time monitoring by the experimenter of the participants’ behavior and progression of the experiment. A standard video camera was also placed, just above the height of the table in front of the participant, for replay in case of technical, kinematic or other issues. Data loss from the Codamotion motion tracking system on a crucial after-effect trial for one of the patients led to analysis performed on the video camera recording (detailed in the legend of corresponding figures).

### Experimental procedure

The experiment consisted of a series of arm reaching movements, performed with either the dominant arm (DA) or the non-dominant arm (NDA), from the starting position toward a visual target. The visual target was flashed 1 s after the beginning of a trial for a short duration of 0.3 s, so that by the time participants had reached the target, it had disappeared. Two auditory tones were then used to inform participants of key timepoints of the trial: a 100-ms-long beep occurring 1.6 s after trial onset to inform participants they could return slowly to the starting location and a 600-ms-long beep occurring 7.4 s after trial onset to inform the participant that the trial had ended. This timing was chosen to allow a slow return movement back to the start button to reduce the impact of the return phase on the adaptation process, as Kitazawa et al. (1997) showed velocity-specific prismatic adaptation. The the return phase was not analyzed within the results. Each trial was 8 s long in total and the next trial started automatically once the previous trial had ended.

Participants were instructed to reach as fast and as accurately as possible toward the visual target in a natural, unconstrained movement. Participants were asked to lift their finger off the table, rather than slide directly across the table and not correct the end position of their finger once it had hit the table. On the return movement, participants were asked to go back slowly to the starting position to minimize the effect of this return phase on the adaptation process. Participants were allowed to return to the start position by sliding their finger along the table. In order to achieve consistent task completion and reduce learning effects during baseline, participants were familiarized with the task by performing 30 reaching movements with both arms under normal visual conditions without prisms before starting the experimental phases. Lastly, participants were instructed not to move their opposite arm during and between trials being performed with the designated arm.

To assess sensorimotor adaptation and interlimb transfer, we employed a procedure inspired by previous work (Harris, 1963; Dizio and Lackner, 1995; Martin et al., 1996a; Kitazawa et al., 1997; Lefumat et al., 2015) and recently used by Renault et al. (2020). The experimental session consisted of three phases (presented in Fig. 2): a baseline preexposure phase under normal vision (baseline phase), a prism exposure phase with prismatic perturbation (prism phase) and a postexposure phase under normal vision (post phase). During the baseline phase, participants performed 30 reaching movements with the DA, then 30 movements with the NDA toward one of the three targets while wearing standard control goggles. The targets were presented in a randomized order which was the same for each participant with, ultimately, 10 trials per target for each arm. The order of experimental conditions in the baseline phase was not counterbalanced, as in other studies (Wang et al., 2011; Lefumat et al., 2015; Renault et al., 2020), because it was desired that all controls and patients performed exactly the same protocol to strengthen control-patient comparisons. When the baseline phase was over, participants had a 2-min break during which they were asked to stay motionless with the eyes closed while the control goggles were replaced with prismatic goggles.

During the following prism phase, participants performed 100 movements (control group A, patient MS and patient AM) or 50 movements (control group B and patient MM) toward the middle 0° target with the DA while wearing the 17° rightward deviating prismatic goggles. Patient MM, and subsequently control group B, completed 50 of the desired 100 movements because of patient MM experiencing tiredness of the right shoulder during this prism phase. The group factor was thus included in the statistical design. At the end of this phase, another 2-min break was given during which participants were instructed to keep their eyes closed and remain motionless, while the prismatic goggles were replaced with the control goggles.

During the post phase, participants first performed 30 reaching movements with the unexposed NDA, before performing 30 movements with the DA again resulting in 10 trials per target per arm under normal vision. During this post phase, the first target presented (post 1 trial) was always the middle straight-ahead 0° target, before all remaining targets were presented in a randomized fashion. The order of experimental phases was selected, as in previous studies (Lefumat et al., 2015; Renault et al., 2020), to have the NDA baseline and post phases immediately before and after the DA prism adaptation phase. Any difference in NDA performance could thus be directly attributed to DA prism adaptation, thus showing interlimb transfer.

Interlimb transfer of sensorimotor adaptation was investigated from DA to NDA based on experimental studies showing unidirectional transfer from the DA to the NDA (Galea et al., 2007; Balitsky Thompson and Henriques, 2010; Mostafa et al., 2014) and, in particular, the study by Criscimagna-Hemminger et al. (2003), which also challenged the role of the corpus callosum in the interlimb transfer of sensorimotor adaptation. Adaptation during the prism phase was performed only toward the middle 0° target so that it would be possible to explore, for both arms, the extent of generalization across target directions in the post phase compared with the baseline phase. This was based on previous literature (Lefumat et al., 2015; Renault et al., 2020), which found significant generalization for the exposed arm but not the unexposed arm. However, to keep the main message of the article clear and not unnecessarily lengthen the manuscript, analysis of the movements toward the lateral targets was not included in the manuscript. Interlimb transfer was thus assessed by comparing baseline movements toward the middle target, performed just before prism adaptation, to the first movement of the post phase toward the middle target, performed just after prism adaptation. This movement was thus performed immediately after prism adaptation and was not influenced by movements to the lateral targets. The experiment took ∼1 h.

### Kinematic data analysis

Data were analyzed using MATLAB (MathWorks) and Microsoft Excel 2017. A few trials (1.8%) had to be discarded because of either the participant not making a movement toward the target, the participant moving before the target had appeared, or technical problems. Position data from the markers on the right and left index fingertips were low-pass filtered with a dual-pass, no-lag Butterworth filter (cutoff frequency: 8 Hz; order: 2). Movement onset and offset were defined as the first time at which hand velocity went above 3 cm/s or dropped below 3 cm/s, respectively (as in Lefumat et al., 2015; Renault et al., 2020). Kinematic variables calculated and reported included: initial movement direction, final movement direction, end point accuracy, maximum perpendicular deviation, peak velocity, time to peak velocity, movement time and reaction time. Initial movement direction was computed as the angle between the vector from the start position to the target position and the vector from the start position to the hand position at peak velocity (Wang and Sainburg, 2003; Renault et al., 2020). Final movement direction was calculated as the angle between the vector from the start position to the target position and the vector from the start position to the hand position at movement offset. End point accuracy was computed as the Euclidian distance in cm between the hand end position and the target position. Maximum perpendicular deviation was calculated as the maximum horizontal (*x*-axis) distance in cm between the movement trajectory path and the theoretical straight line connecting the start position and the target position (Shadmehr and Moussavi, 2000; Malfait and Ostry, 2004).

The kinematic variable of interest for examining the prismatic effects throughout the experiment was the initial movement direction as this mostly reflects the initial motor plan before visual feedback loops influence the movement (O’Shea et al., 2014; Reichenbach et al., 2014; Sarlegna and Mutha, 2015). Maximum perpendicular deviation was also reported to verify prismatic adaptation and transfer effects, noted as giving similar results by Malfait and Ostry (2004).

### Statistical data analysis

R3.6.0 (R Core Team, 2018), Statistica 8 (StatSoft), and Excel 2017 were used to perform statistical analysis. Statistica was used to assess normal distribution with the Kolmogorov–Smirnov method, perform *t* tests and ANOVAs, and carry out Tukey *post hoc* analysis of control data. Excel 2017 was used to calculate individual 98% confidence interval boundaries for both controls and patients, using individual participant’s own baseline data. Confidence intervals were constructed for the normally distributed data using confidence interval formula including the mean ($\overset{¯}{x}$), two-tailed *t* value SD (s), and sample size (*n*). A two-tailed design at 98% confidence was used to test for deviation in either direction with an *α*/2 of 0.01 (*p *<* *0.02) and *t* values were used because of a small sample size of baseline trials (*n *< 30; Moore et al., 2009; Pek et al., 2017), with 10 trials per target per arm. R using parts of the *psycho* (v0.5.0; Makowski, 2018) package, was used to perform Crawford’s modified *t* test. This method, adapting an independent sample pooled *t* test for use with a sample of *n *=* *1 (one patient), was used to compare each patient’s performance to that of a control sample (Crawford and Garthwaite, 2007). Results were compared with a Bayesian method using the software Single_Bayes_ES, with similar results obtained (Crawford et al., 2010). *Z* values were reported as an indicator of effect size. The Kolmogorov–Smirnov method showed all data to be normally distributed.

Analysis of control group baseline kinematics consisted of 2 × 2 ANOVAs including the two groups: group A and group B and two arms (repeated measures): DA arm and NDA arm. The factor group (wo groups: group A: age = 52 ± 4 years, 100 trials, *n *=* *8 and group B: age = 29 ± 4 years, 50 trials, *n *=* *8) was included within all analyses to check for putative effects. Kinematic variables assessed included: initial movement direction, final movement direction, end point accuracy, maximum perpendicular deviation, peak velocity, time to peak velocity, movement time, and reaction time. Patient values were then compared with the control group for each patient, across each arm individually, using Crawford’s modified *t* test.

Analysis of controls’ DA adaptation consisted of a 2 × 16 ANOVA on initial movement direction including the two groups: group A and group B, and 16 DA phases (repeated measures): baseline 10 trial average, prism trials 1, 2, 3, 4, 5, 6, 7, 8, 9, 10, prism 11–20 10 trial average, prism 21–30 average, prism 31–40 average, and the prism 41–50 last common average, as well as the post 1 trial. On an individual level, including both controls and patients, prismatic effects and after-effects according to initial movement direction and maximum perpendicular deviation were explored by comparing specific trials (prism phase trials and the post 1 trial, respectively) to the individual’s baseline 98% confidence interval. Trials falling above or below the baseline 98% confidence interval boundaries were deemed to be significantly different to baseline. The number of trials for each participant to reduce errors caused by the prismatic perturbation (error-reduction rate) was taken as the first prism phase trial to return within the 98% baseline confidence interval. The prismatic-effect and after-effect for the DA of each individual were then quantified by calculating the difference between the baseline phase average and the prism 1 and post 1 values, respectively. Patient prismatic-effects, error-reduction rates, and after-effects were then compared with the control group average using Crawford’s modified *t* test.

Analysis of control group NDA data exploring interlimb transfer effects consisted of a 2 × 2 ANOVA on initial movement direction data including the two groups: group A and group B and the two phases (repeated measures): baseline 10 trial average and post 1. For each individual, the NDA post 1 trial was compared with the baseline 98% confidence intervals to determine the presence of interlimb transfer according to both initial movement direction and maximum perpendicular deviation. A post 1 trial falling above or below the baseline 98% confidence interval boundary was deemed to be significantly different compared with baseline, thus showing interlimb transfer. The interlimb transfer value was then quantified for each individual as the difference between the baseline value and the post 1 value and transformed into an absolute value to compare the amplitude of transfer without directional effects. Patients’ transfer-effects were then compared with the control group average using Crawford’s modified *t* test. For control group and patient-control comparisons, the significance threshold was set to 0.05.

The ANOVAs performed on controls’ data included 10-trial averages as well as individual trials, in line with previous research (Morton and Bastian, 2004; Taylor et al., 2011; Lefumat et al., 2015; Leclere et al., 2019; Renault et al., 2020). This was because, in the current study, data analyses revealed some blocks of trials with homogenous performance and blocks of trials with variable performance. Averaging trials thus made sense when motor performance was stable and homogenous, as in baseline and late prism trials, to have a better estimate of performance. However, when large variations were observed between consecutive trials, such as during the initial prism error-reduction phase and post phase, individual trials were kept separate to avoid masking an effect such as interlimb transfer (Taylor et al., 2011).

## Results

### Baseline motor control

**Figure 3.**
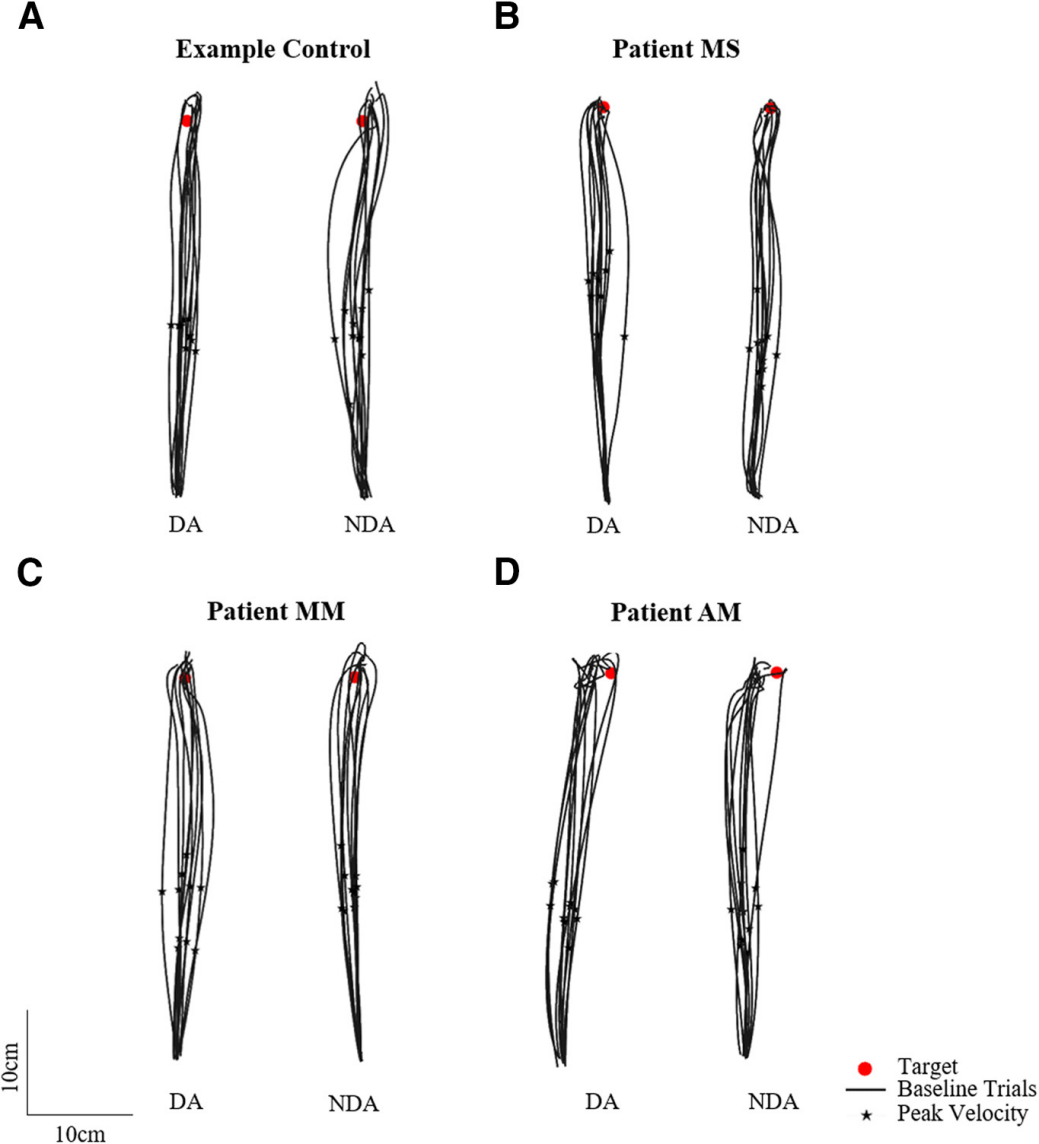
Baseline phase top-down view of the 10 reaching hand paths toward the middle straight-ahead target (red circle) for the dominant arm (DA) and non-dominant arm (NDA) for (***A***) an example control, (***B***) patient MS, (***C***) patient MM, and (***D***) patient AM. Peak velocity is indicated with a black star.

Participants were asked to reach as fast and as accurately as possible toward visual targets with either the DA or NDA arm, under normal visual conditions with visual feedback of the arm at all times. Figure 3 shows baseline trajectories toward the straight-ahead target for an example control participant and three neurologic patients with corpus callosum abnormalities. Figure 3 shows that controls, patients MS and MM, whose corpus callosum was severed by a stroke, as well as patient AM, who has a complete corpus callosum agenesis, were able to reach to the target. Hand path trajectories for patients and controls seemed comparably straight and accurate, suggesting that the callosal patients had a normal spatial organization of the movements.

**Figure 4.**
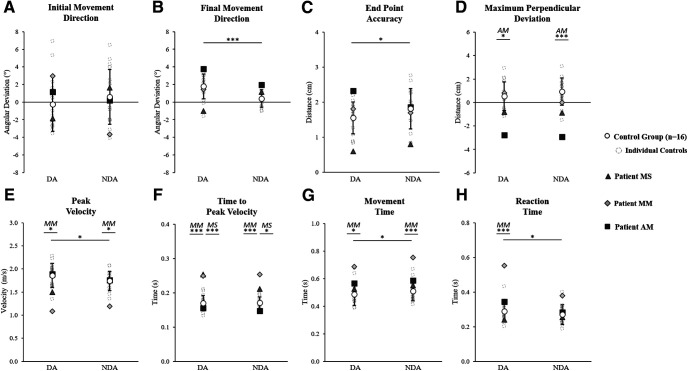
Baseline kinematics for the dominant arm (DA) and non-dominant arm (NDA) movements to the middle straight-ahead target. ***A***, Initial movement direction. ***B***, Final movement direction. ***C***, End point accuracy. ***D***, Max perpendicular deviation. ***E***, Peak velocity. ***F***, Time to peak velocity. ***G***, Movement time. ***H***, Reaction time. Data are shown for the control group (*n* =* *16) average (white circles with SD error bars) and individual average values for controls (gray dashed circles), patient MS (triangles), patient MM (diamonds), and patient AM (squares). Significant differences between the control group DA and NDA, according to a 2 × 2 (arm × group) ANOVA, are marked with spanning black asterisks. For each arm, significant differences between a patient and the control group, according to Crawford’s modified *t* test, are indicated by black asterisks with corresponding patient initials (MS, patient MS; MM, patient MM; AM, patient AM); *p < 0.05, ***p < 0.01.

Control participants’ baseline data were analyzed with a mixed factor 2 × 2 ANOVA including two arms (DA and NDA) and two groups (group A and group B). Interlimb differences were found on certain control group kinematics (Fig. 4) as the ANOVA showed a simple arm effect for final movement direction (controls average ± SD: DA = 1.8 ± 1.4°, NDA = 0.4 ± 1.0°; *F*_(1,14)_ = 17.0; η_p_^2^ = 0.55, *p* = 0.001), end point accuracy (DA = 1.5 ± 0.5 cm, NDA = 1.8 ± 0.6 cm; *F*_(1,14)_ = 4.6; η_p_^2^ = 0.25, *p* = 0.049), peak velocity (DA = 1.9 ± 0.3 m/s, NDA = 1.7 ± 0.2 m/s; *F*_(1,14)_ = 7.9; η_p_^2^ = 0.36, *p* = 0.014), movement time (DA = 486 ± 80 ms, NDA = 510 ± 70 ms; *F*_(1,14)_ = 8.0; η_p_^2^ = 0.37, *p* = 0.013), and reaction time (DA = 289 ± 58 ms, NDA = 270 ± 57 ms; *F*_(1,14)_ = 8.5; η_p_^2^ = 0.38, *p* = 0.011). There were no significant group effects nor interactions. Patient values were thus compared with the whole control group (*n* = 16).

Each patient’s baseline average was compared with the controls using Crawford’s modified *t* test for each kinematic variable and each arm individually. This analysis showed that the motor performance of patient AM significantly differed from controls only on maximum perpendicular deviation with a more leftward deviation than controls for both the DA (controls = 0.5 ± 1.2 cm, AM = −2.7 ± 0.7 cm; *z* = −2.71, *p* = 0.019) and NDA (controls = 0.9 ± 1.2 cm, AM = −2.9 ± 0.9 cm; *z* = −3.30, *p* = 0.006; Fig. 4*D*). Patient MS showed one significant difference with a longer time to peak velocity than controls, for both the DA (controls = 170 ± 22 ms, MS = 255 ± 34 ms; *z* = 3.90, *p* = 0.002) and NDA (controls = 171 ± 17 ms, MS = 211 ± 37 ms; *z* = 2.34, *p* = 0.039; Fig. 4*F*). Patient MM, tested the soonest after corpus callosum insult, showed a reduced peak velocity for both the DA (controls = 1.9 ± 0.3 m/s, MM = 1.1 ± 0.1 m/s; *z* = −2.96, *p* = 0.011) and NDA (controls = 1.7 ± 0.2 m/s, MM = 1.2 ± 0.2 m/s; *z* = −2.68, *p* = 0.020; Fig. 4*E*), a longer time to peak velocity for both the DA (controls = 170 ± 22 ms, MM = 249 ± 55 ms; *z* = 3.62, *p* = 0.003) and NDA (controls = 171 ± 17 ms, MM = 255 ± 2 ms; *z* = 4.88, *p* < 0.001; Fig. 4*F*) and a longer movement time for both the DA (controls = 486 ± 80 ms, MM = 686 ± 41 ms; *z* = 2.49, *p* = 0.028) and NDA (controls = 510 ± 70 ms, MM = 754 ± 56 ms; *z* = 3.48, *p* = 0.004). Patient MM also exhibited a longer reaction time for the DA (controls = 289 ± 58 ms, MM = 553 ± 194 ms, *z* = 0.0005, *p* = 0.018) but not the NDA (controls = 270 ± 57 ms, MM = 379 ± 95 ms; *z* = 1.91, *p* = 0.083; Fig. 4*H*) indicating a larger arm effect than the control group for this variable. Overall, baseline results show that all patients were able to reach accurately toward the visual target when considering initial and final errors: some temporal differences were observed but the spatial organization of the movements was comparable between the patients and controls.

### Prismatic adaptation of the DA arm

To assess sensorimotor adaptation with the DA, participants were asked to perform reaching movements with the DA before (baseline phase), during (prism phase), and after (post phase) prismatic exposure. For controls (*n* = 16), a 2 × 16 ANOVA of peak velocity showed no significant group effect (*F*_(1,14)_ = 1.78, η_p_^2^ = 0.11, *p* = 0.203) or interaction (*F*_(15,210)_ = 1.20, η_p_^2^ = 0.08, *p* = 0.275) and a significant effect of phase (*F*_(15,210)_ = 2.25, η_p_^2^ = 0.14, *p* = 0.006). Tukey *post hoc* analysis showed that the phase effect was because of an augmented peak velocity on prism 1 (2.0 ± 0.4 m/s) compared with subsequent prism trials 4, 6, 7, 8, 9, and 10, with peak velocities in the range of 1.7–1.8 m/s (*p* range < 0.001–0.046). No significant differences were observed between baseline peak velocity (1.9 ± 0.3 m/s) and any of the subsequent prism phases (combined peak velocity = 1.8 ± 0.3 m/s, all *p*s > 0.49) or the post 1 trial (1.8 ± 0.4 m/s, *p* > 0.99). For patient MS, peak velocity did not significantly differ from controls (*n* = 16) in any phase. Patient AM showed few significant differences compared with controls with an increased peak velocity on prism 1 (controls = 2.0 ± 0.4 m/s, AM = 3.1 m/s; *z* = 2.40, *p* = 0.035) and prism 5 (controls = 1.8 ± 0.2 m/s, AM = 2.4 m/s; *z* = 2.61, *p* = 0.023). For patient MM, peak velocity was significantly lower than controls (*n* = 16) across all adaptation phases (MM range: 0.8–1.1 m/s, −3.35 < *z* < −2.52, 0.006 < *p* < 0.045) with the exception of prism 1 (controls = 2.0 ± 0.4 m/s, MM = 1.2 m/s; *z* = −2.03, *p* = 0.069) and prism 6 (controls = 1.7 ± 0.2 m/s, MM = 1.2 m/s; *z* = −1.86, *p* = 0.090). Overall, movement speed was relatively constant for each patient, with patients MS and AM having no or few significant differences in peak velocity compared with controls, while patient MM showed reduced peak velocity.

**Figure 5.**
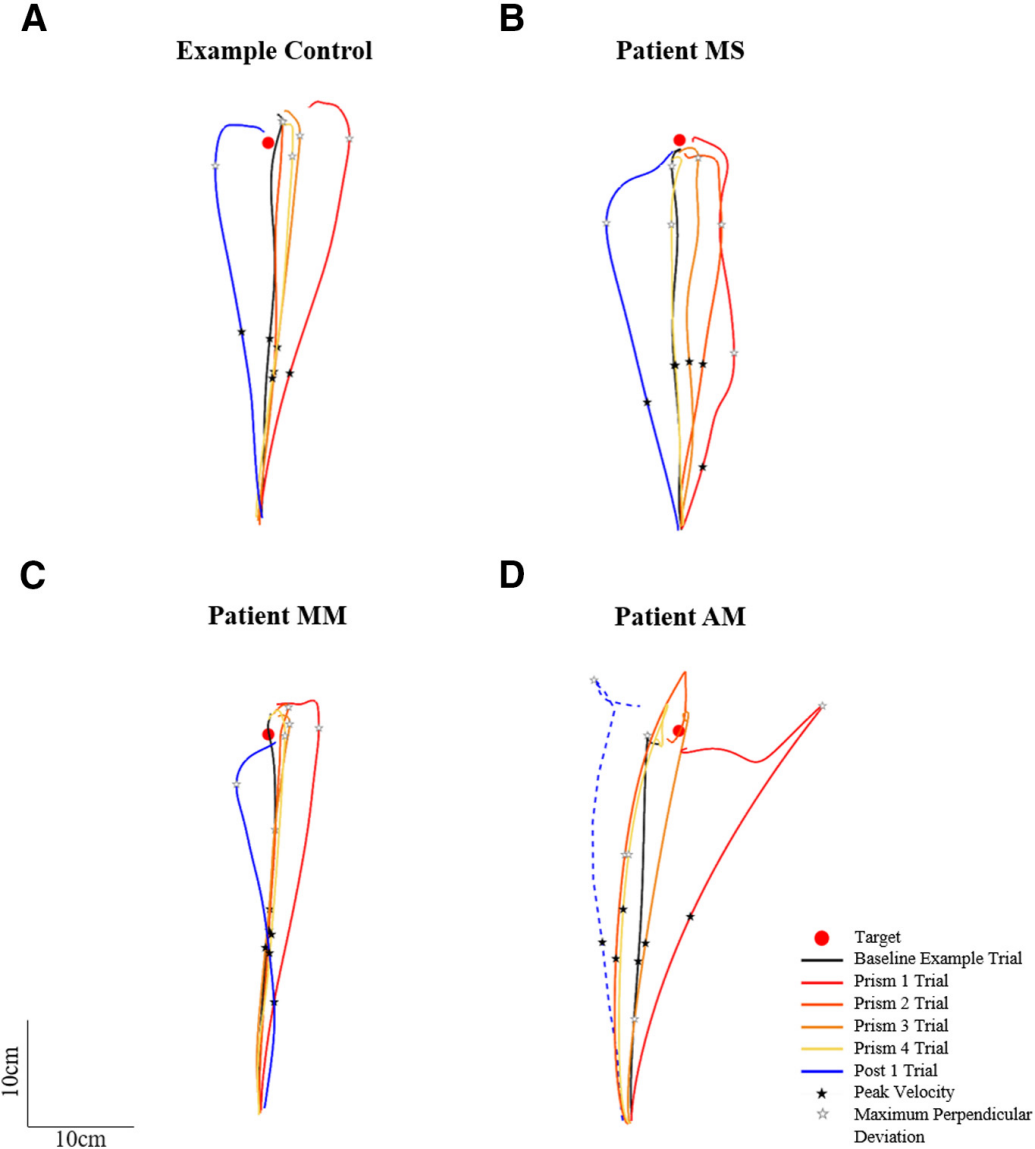
Prism-exposed dominant arm top-down view of hand paths toward the target (red circle) for (***A***) an example control, (***B***) patient MS, (***C***) patient MM, and (***D***) patient AM. Trajectories include: a baseline phase representative trial (black), prism trials 1 (red), 2 (dark orange), 3 (light orange), and 4 (yellow), and the post 1 trial (blue). The blue dashed line in panel ***D*** is the estimated post 1 trial trajectory for patient AM calculated based on motion tracking of a standard video camera recording using ImageJ manual tracking software and adjustment according to a standard baseline velocity profile, as a technical issue on this trial caused kinematic data loss via the Codamotion system. Occurrence of peak velocity for each trial is marked with a black star; occurrence of maximum perpendicular deviation is marked with a white star.

**Figure 6.**
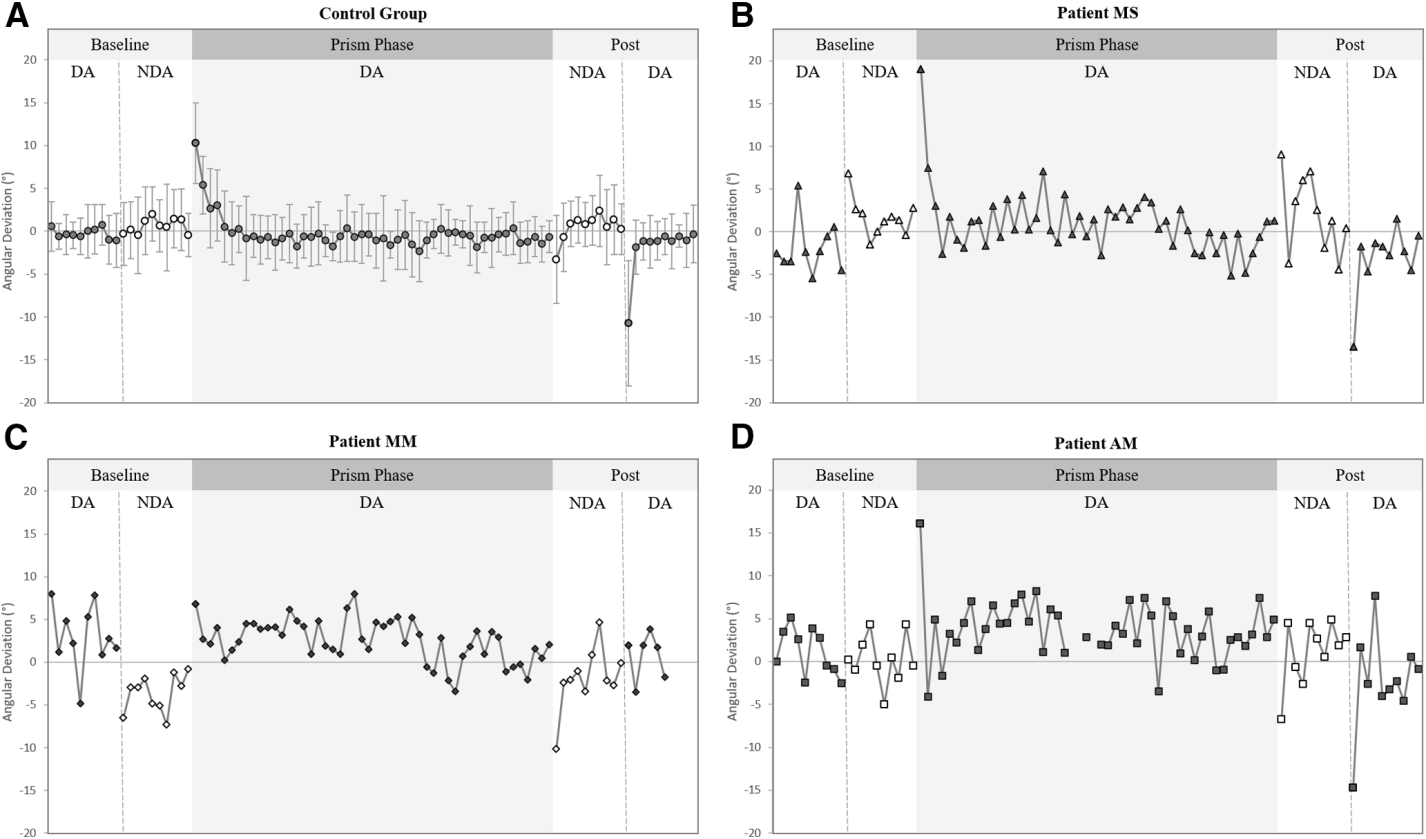
Initial movement direction for both the dominant arm (DA, represented as black filled symbols) and non-dominant arm (NDA, represented as white filled symbols) across movements toward the middle target for (***A***) the control group (*n* = 16) average values (circles), (***B***) patient MS (triangles), (***C***) patient MM diamonds), and (***D***) patient AM (squares). Data shown include: all 10 baseline trials toward the middle target (DA then NDA), prism trials 1–50 toward the middle target (DA only), and all 10 post trials toward the middle target (NDA then DA). Error bars in panel ***A*** represent SDs of the control group mean. The post 1 value for patient AM in panel ***D*** was calculated from an estimated trajectory created using ImageJ motion tracking of a standard videorecording and adjustment according to a standard baseline velocity profile, as Codamotion kinematic data were lost because of a technical issue on this trial.

**Figure 7.**
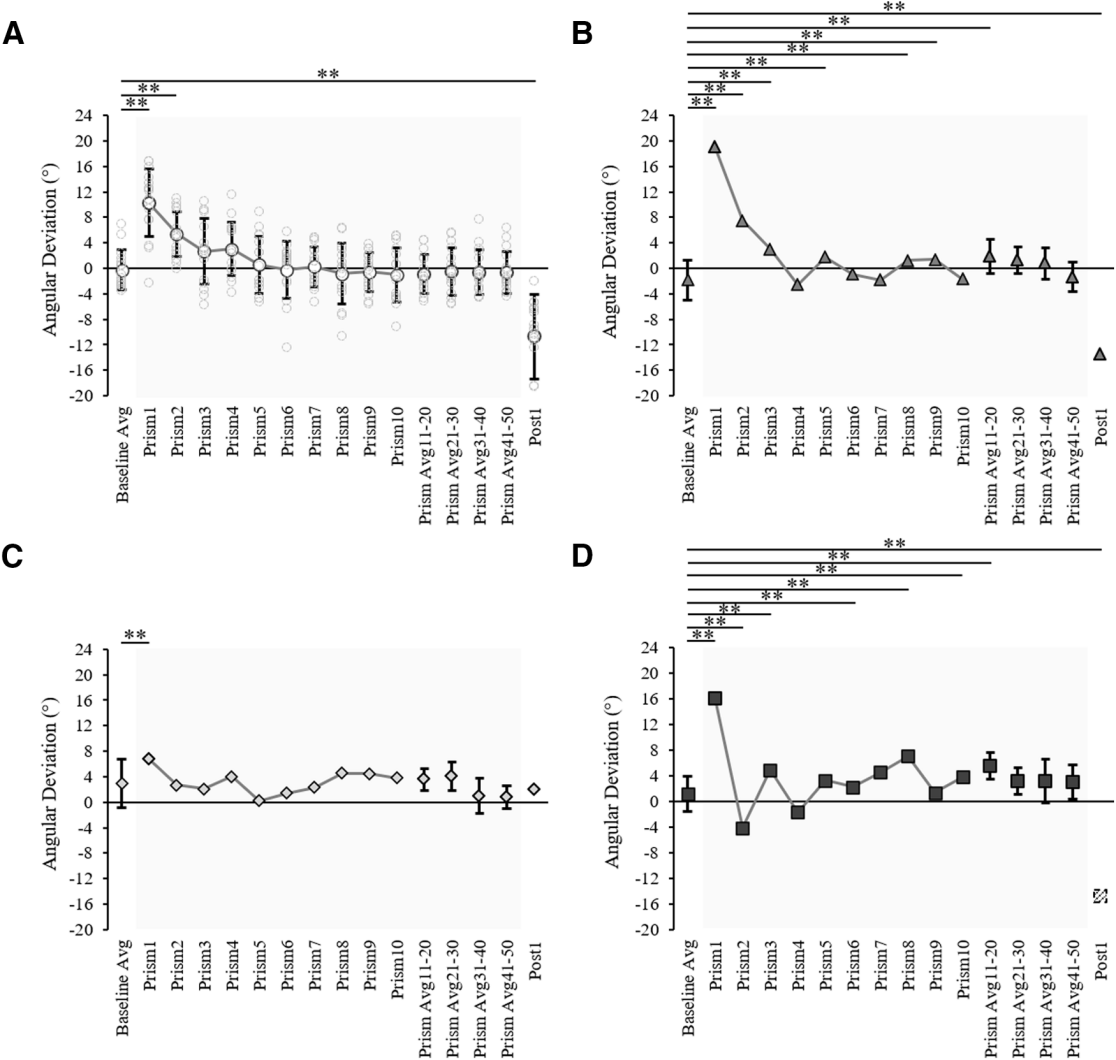
Prism-exposed DA arm initial movement direction across trials for (***A***) the control group (*n* =* *16) showing group average (white circles) and individual values (light gray circles), (***B***) patient MS (gray triangles), (***C***) patient MM (light gray diamonds), and (***D***) patient AM (dark gray squares). Data shown include: baseline (10 trial average), prism trials 1–10, the last 10 prism trials average (prism 41–50), and the post 1 trial. Error bars in panel ***A*** represent control group SDs, asterisks indicate trials which significantly differ to baseline according to a 2 × 16 (group × phase) ANOVA. Error bars in panels ***B–D*** represent the individual patient SDs for baseline (10 trials) and the last common prism phase (10 trials), asterisks indicate trials which significantly differ from the baseline average according to baseline 98% confidence interval analysis. All asterisks are indicated at the threshold **p < 0.02. The post 1 value for patient AM in panel ***D*** was calculated from an estimated trajectory created using ImageJ motion tracking of a standard videorecording and adjustment according to a standard baseline velocity profile, as Codamotion kinematic data were lost because of a technical issue on this trial.

**Figure 8.**
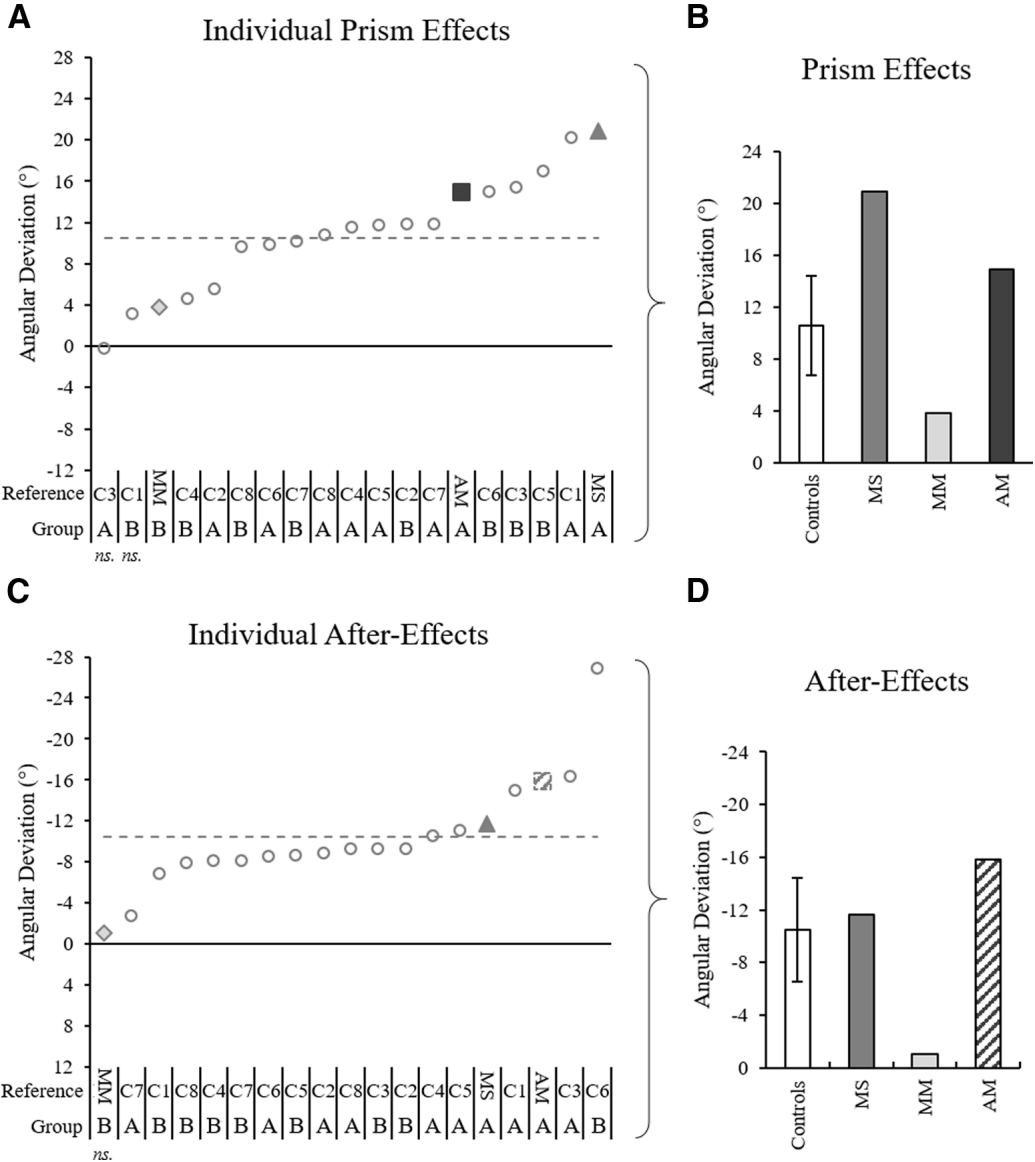
Prismatic effects and after-effects for each individual, quantified with initial movement direction analysis. ***A***, ***C***, Initial movement direction across trials for all individual controls (gray circles), patient MS (gray triangle), patient MM (light gray diamond), and patient AM (dark gray square), calculated as the difference between each individual’s baseline average and the individual’s prism 1 or post 1 trial, respectively. Notations below the graphs indicate patient initials (MM, MS, MM) and control references (C1–C8) for each corresponding group (group A: 52 ± 4 years old, 100 prism trials; group B: 29 ± 4 years old, 50 prism trials). The gray dashed lines mark the control group average. ***B***, ***D***, Data in panels ***A***, ***C***, respectively, with control data represented by the control group average and SD. The post 1 value for patient AM in panels ***C***, ***D*** was calculated from an estimated trajectory created using ImageJ motion tracking of a standard videorecording and adjustment according to a standard baseline velocity profile, as Codamotion kinematic data were lost because of a technical issue on this trial.

Spatial hand paths of the DA showing prismatic effects can be seen in Figure 5. From this, it can be seen that when control participants (Fig. 5*A*) and patients (Fig. 5*B–D*) wore rightward-deviating prisms, the first trial with the prisms (prism 1) was deviated rightward compared with baseline, often with late online corrections toward the target. This also appears on Figure 6, which shows initial movement direction for each experimental trial. The ANOVA of controls’ initial movement direction showed no significant group effect (*F*_(1,14)_ = 0.75, η_p_^2^ = 0.05, *p* = 0.402) or interaction (*F*_(15,210)_ =1.13, η_p_^2^ = 0.08, *p* = 0.332), and a significant effect of phase (*F*_(15,210)_ = 35.5, η_p_^2^ = 0.72, *p* < 0.001) with Tukey *post hoc* analysis revealing significant deviations on prism 1 compared with baseline (baseline = −0.2 ± 3.1°, prism 1 = 10.3 ± 5.3°, *p* < 0.001; Fig. 7*A*). Individual 98% confidence interval analysis on initial movement direction showed rightward deviation on prism 1 for 14/16 controls with 2/16 controls not significantly deviated. The same 98% confidence interval analysis revealed significant deviation for patient MS (baseline = −1.9 ± 3.1°, 98% CI [−4.6, 0.9], prism 1 = 19.0°; Fig. 7*B*), patient MM (baseline = 3.0 ± 3.8°, 98% CI [−0.4, 6.4], prism 1 = 6.8°; Fig. 7*C*), and patient AM (baseline = −1.1 ± 2.8°, 98% CI [−1.3, 3.6], prism 1 = 16.1°; Fig. 7*D*). All individuals’ quantified prism effects (prism 1, baseline) are shown in Figure 8*A*. Crawford’s modified *t* test on the prism effect showed no significant differences between controls (*n* = 16; 10.5 ± 5.3°) and patient MS (MS = 20.9°, *z* = 1.95, *p* = 0.078), patient MM (MM = 3.8°, *z* = −1.27, *p* = 0.239), or patient AM (AM = 14.9°, *z* = 0.83, *p* = 0.433; Fig. 8*B*).

**Figure 9.**
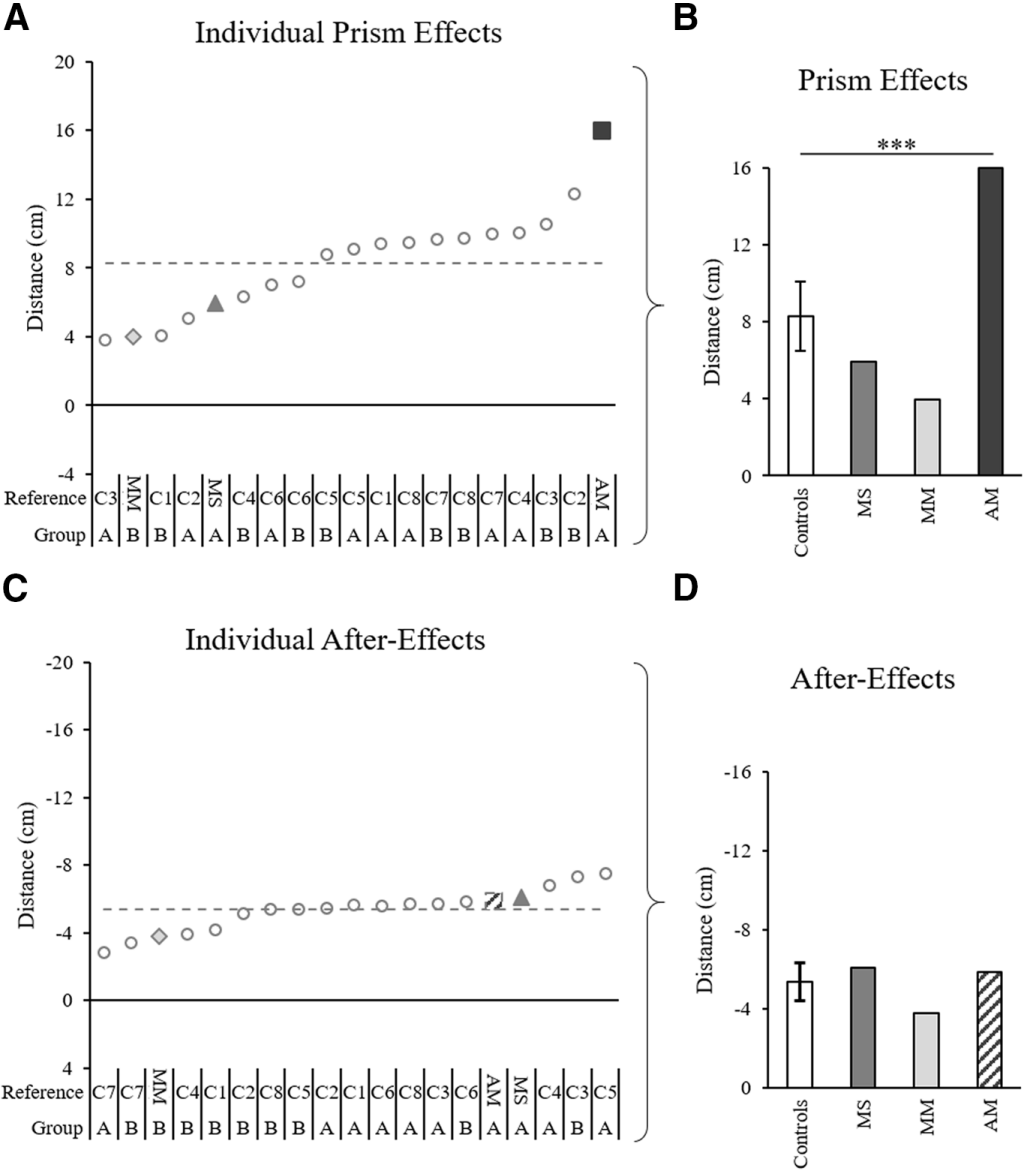
Prism effects and after-effects for each individual, quantified based on maximum perpendicular deviation analysis. ***A***, ***C*,** Quantified effect values according to maximum perpendicular deviation across all individual controls (gray circles), patient MS (gray triangle), patient MM (light gray diamond), and patient AM (dark gray square), calculated as the difference between each individual’s exposed DA arm baseline average and prism 1 or post 1 trial, respectively. Notations below the graphs indicate patient initials (MM, MS, MM) and control references (C1–C8) for each corresponding group (group A: 52 ± 4 years old, 100 prism trials; group B: 29 ± 4 years old, 50 prism trials). The gray dashed lines mark the control group average. ***B***, ***D***, Data in panels ***A***, ***C***, respectively, with control data represented by the control group average and SD. Asterisks in panels ***B***, ***D*** indicated significant differences between the patients and the control group according to Crawford’s modified *t* test; ***p < 0.01. The post 1 value for patient AM in panels ***C***, ***D*** was calculated from an estimated trajectory created using ImageJ motion tracking of a standard videorecording and adjustment according to a standard baseline velocity profile, as Codamotion kinematic data were lost because of a technical issue on this trial.

The analysis of maximum perpendicular deviation provided further evidence for patients and controls having typical prismatic effects. Individual 98% confidence interval analysis showed that on prism 1, 16/16 controls, and all three patients were significantly deviated rightward by the prisms compared with baseline (for individuals’ prism effects, see Fig. 9*A*). According to Crawford’s modified *t* test, there were no significant differences between the controls’ (*n* = 16) prism effect (controls = 8.3 ± 2.5 cm) and patient MS (MS = 5.9 cm, *z* = 0.37, *p* = 0.410) or patient MM (MM = 4.0 cm, *z* = −1.76, *p* = 0.116), while patient AM had a larger prismatic effect than controls (AM = 16.0 cm, *z* = 3.16, *p* = 0.008; Fig. 9*B*).

A classic pattern of error reduction was then observed following the first prism trial with less deviated trajectories on prism trials 2, 3, and 4 (Fig. 5). Results from the control group ANOVA on initial movement direction showed a maintained significant deviation on prism 2 compared with baseline (baseline = −0.2 ± 3.1°, prism 2 = 5.4 ± 3.6°, *p* < 0.001) with this deviation no longer significant on prism 3 (2.7 ± 5.2°, *p* = 0.212). On an individual level, 14 of the 14 controls perturbed by the prisms on prism 1 were still perturbed on prism 2, eight controls on prism 3, and six controls on prism 4. The number of trials to correct the prismatic perturbation and reduce errors, taken as the first trial to fall within the 98% baseline confidence intervals, was 4.5 ± 2.6 trials (range = 3–9 prism trials) on average for controls. Patient MS reduced errors by prism trial 4 (Fig. 7*B*), patient MM by prism 2 (Fig. 7*C*), and patient AM by prism 5 (Fig. 7*D*). Crawford’s modified *t* test showed no significant difference in the number of trials to reduce errors between controls (*n* = 16) and patients (controls = 4.5 ± 2.6 trials; MS = 4 trials, *z* = −0.19, *p* = 0.855; MM = 2 trials, *z* = −0.96, *p* = 0.366; AM = 5 trials, *z* = 0.19, *p* = 0.854).

Typical leftward deviated trajectories indicating an after-effect were then apparent on the first post movement (post 1) with the DA, despite this trial occurring after the NDA post phase of 30 trials (Fig. 5). For the control group (*n* = 16), an ANOVA on initial movement direction showed that the post 1 trial was significantly deviated compared with baseline (baseline = −0.2 ± 3.1°, post 1 = −10.7 ± 6.6°, *p* < 0.001; Fig. 7*A*). Individual 98% confidence interval analysis showed significant deviation on post 1 for 16/16 controls, patient MS (baseline = −1.9 ± 3.1°, 98% CI [−4.6, 0.9], post 1 = −13.5°; Fig. 7*B*) and patient AM (baseline = −1.1 ± 2.8°, 98% CI [−1.3, 3.6], post 1 = −14.7°; Fig. 7*D*). The after-effect for patient MM (Fig. 5*C*) was not significant when analyzing initial movement direction (baseline = 3.0 ± 3.8°, 98% CI [−0.4, 6.4], post 1 = 1.9°; Fig. 7*C*). All individuals’ after-effects (post 1− baseline) are shown in Figure 8*C*. Comparison of the patients’ after-effects to controls (*n* = 16; −10.5 ± 5.3°) using Crawford’s modified *t* test showed no significant differences for patient MS (MS = −11.7°, *z* = −0.22, *p* = 0.789), patient MM (MM = −1.0°, *z* = 1.77, *p* = 0.106) or patient AM (AM = −15.9°, *z* = −1.01, *p* = 0.345; Fig. 8*D*).

Analysis of maximum perpendicular deviation provided results consistent with those of the previous analysis of initial movement direction, with the exception that the after-effect of patient MM was significant. Individual 98% confidence interval analysis of post 1 compared with baseline showed significant after-effects for 16/16 controls and all three patients (for individuals’ after-effects, see Fig. 9*C*). Crawford’s modified *t* test showed that there were no significant differences in the after-effect according to maximum perpendicular deviation between the controls (*n* = 16; controls = −5.4 ± 1.3 cm), patient MS (MS = −6.1 cm, *z* = −0.55, *p* = 0.596), patient MM (MM = −3.8 cm, *z* = 1.22, *p* = 0.255), or patient AM (AM = −5.9 cm, *z* = −0.40, *p* = 0.693; Fig. 9*D*). Overall, these results indicate that all controls and all three patients were deviated rightward by the prisms, showed a typical pattern of error reduction during prism exposure and had characteristic leftward deviating after-effects.

### Transfer of prism adaptation to the NDA arm

Interlimb transfer of sensorimotor adaptation was assessed by comparing reaching movements performed with the NDA immediately before (baseline phase) and immediately after (post 1 trial) the prism phase performed with the DA. For controls (*n* = 16), a 2 × 2 ANOVA on peak velocity including two groups and two phases showed that peak velocity did not significantly differ across the different phases (baseline average = 1.7 ± 0.2 m/s, post 1 = 1.8 ± 0.2 m/s, *F*_(1,14)_ = 0.28, η_p_^2^ = 0.02, *p* = 0.608). No significant group effect (*F*_(1,14)_ = 0.57, η_p_^2^ = 0.04, *p* = 0.462) or interaction (*F*_(1,14)_ = 0.60, η_p_^2^ = 0.04, *p* = 0.453) were found. Comparison of NDA peak velocities on post 1 between controls (*n* = 16) and patients showed that patient MS had no significant difference in peak velocity compared with controls, patient AM had increased peak velocity and patient MM had reduced peak velocity (controls = 1.8 ± 0.2 m/s; MS = 1.6 m/s; *z* = −0.87, *p* = 0.407; AM = 2.3 m/s, *z* = 2.81, *p* = 0.016; MM = 0.9 m/s, *z* = −4.25, *p* < 0.001), consistent with previously reported results.

**Figure 10.**
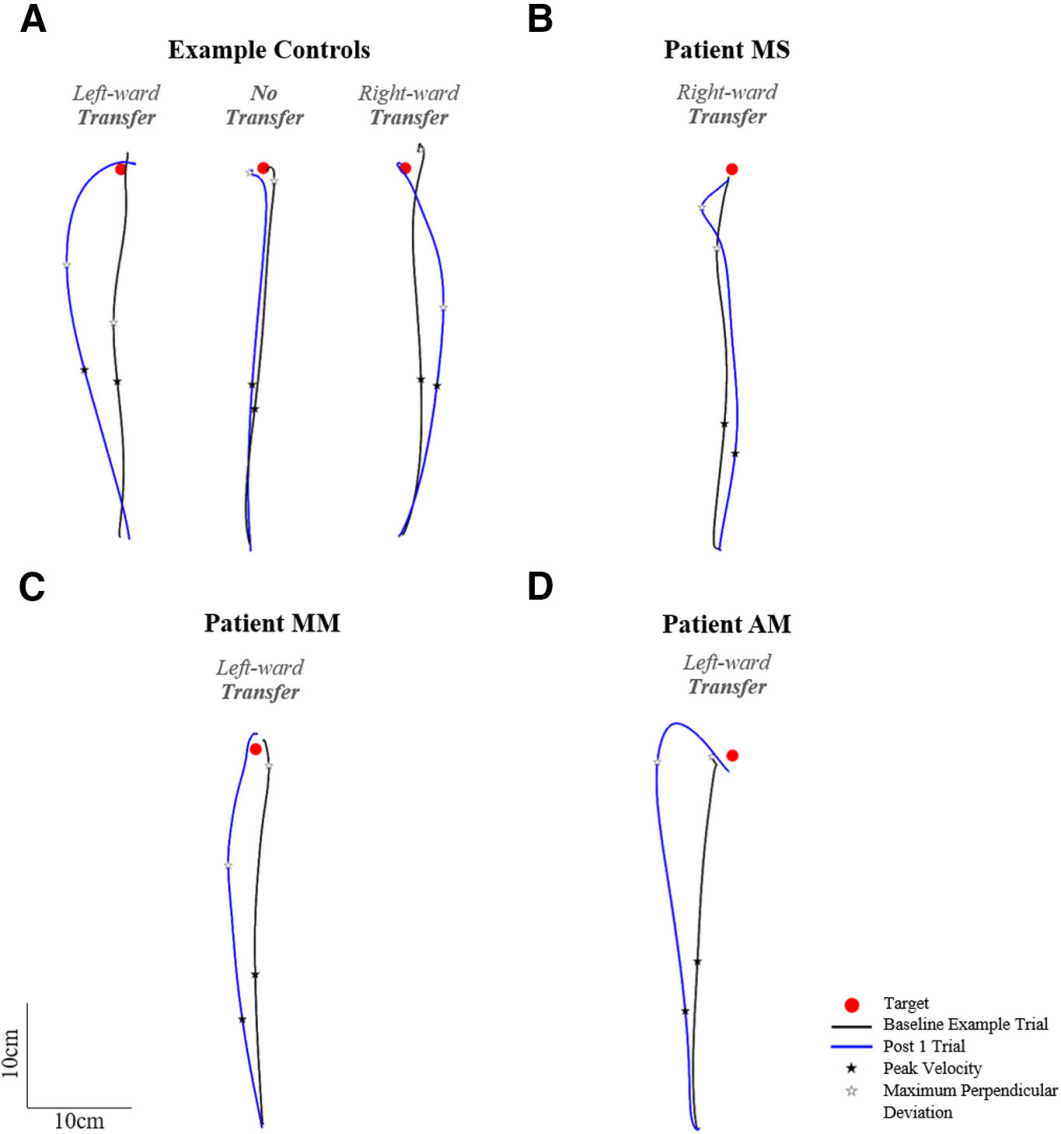
Naive non-dominant arm (NDA) top-view of hand paths for a baseline representative trial (black) and the post 1 trial (blue) for (***A***) three example controls showing leftward, rightward, or no initial deviation on the post 1 trial compared with baseline, (***B***) patient MS showing an initial rightward deviation, (***C***) patient MM showing an initial leftward deviation, and (***D***) patient AM showing an initial leftward deviation. Occurrence of peak velocity is marked with a black star; occurrence of maximum perpendicular deviation is marked with a white star.

**Figure 11.**
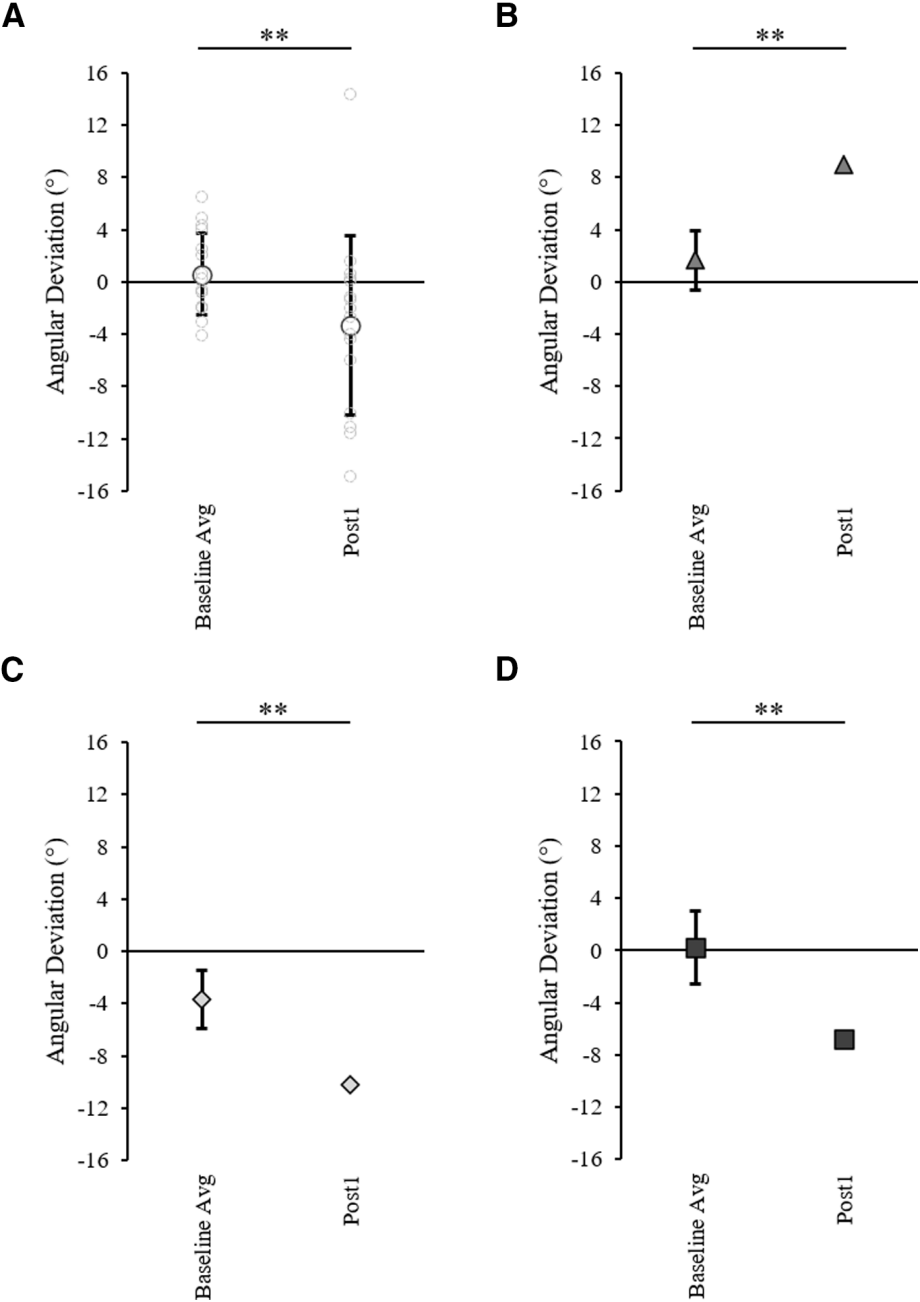
Naive non-dominant arm (NDA) initial movement direction before and after prismatic adaptation with the dominant arm for (***A***) the control group (*n* =* *16) showing the group average (white circles) and individual values (gray circles), (***B***) patient MS (gray triangles), (***C***) patient MM (light gray diamonds), and (***D***) patient AM (dark gray squares). Data show the baseline 10 trial average and post 1 trial. Error bars in panel ***A*** represent control group SDs, and asterisks indicate trials which significantly differ to baseline according to a 2 × 2 (group × phase) ANOVA. Error bars in panels ***B–D*** represent each patient’s baseline SDs, and asterisks indicate trials which significantly differ from the baseline average according to baseline 98% confidence interval analysis. Significance is shown at **p < 0.02 threshold.

**Figure 12.**
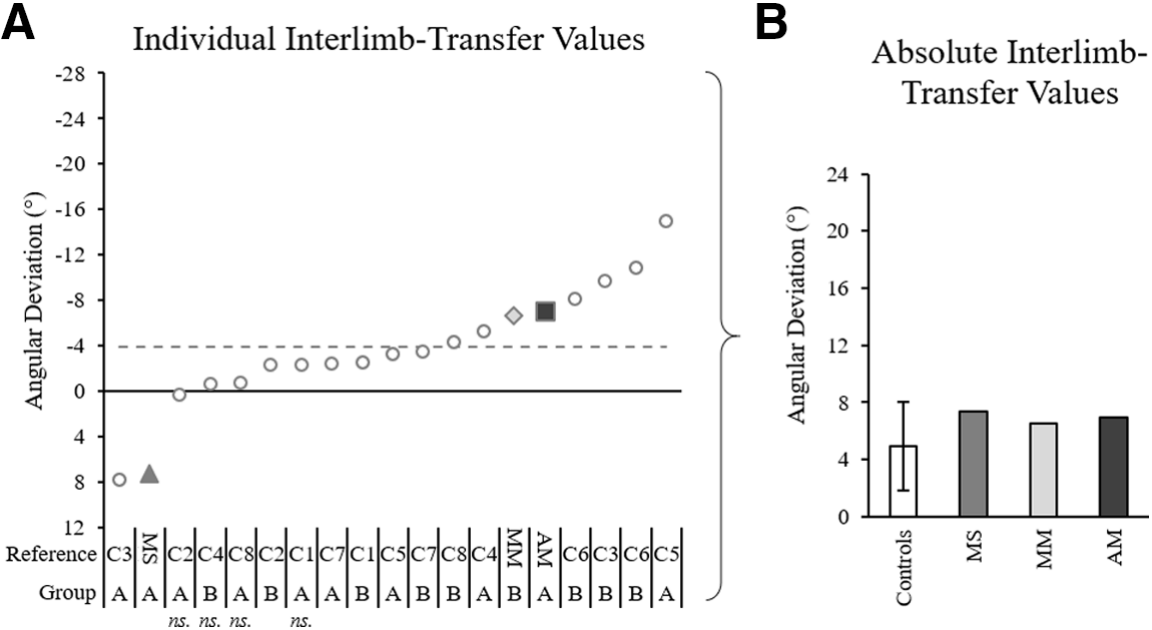
Interlimb transfer for each individual, quantified with initial movement direction analysis. ***A***, Interlimb transfer values according to analysis of initial movement direction for all individual controls (gray circles), patient MS (gray triangle), patient MM (light gray diamond), and patient AM (dark gray square), calculated as the difference between each individual’s naive NDA arm baseline average and post 1 trial. Notations below the graphs indicate patient initials (MM, MS, MM) and control references (C1–C8) for each corresponding group (group A: 52 ± 4 years old, 100 prism trials; group B: 29 ± 4 years old, 50 prism trials). The gray dashed lines mark the control group average; ns. indicates individuals for whom the effect was not significant according to the individual’s baseline 98% confidence interval analysis. ***B***, Absolute transformation of the data in panel ***A*** with control data represented by the control group average and SD.

Figure 10 shows NDA trajectories for three example controls (Fig. 10*A*) and the three patients (Fig. 10*B–D*). Figure 10 shows that the post 1 movement of the NDA appeared deviated compared with the baseline trajectory for the majority of controls as well as patients, with three apparent patterns of transfer: initial rightward deviation, initial leftward deviation or no transfer. A 2 × 2 ANOVA on initial movement direction including two groups and two phases (baseline average and post 1) showed a significant effect of phase, with the post 1 initial movement direction significantly differing from baseline (baseline average = 0.6 ± 3.1°, post 1 = −3.3 ± 6.9°, *F*_(1,14)_ = 9.53, η_p_^2^ = 0.40, *p* = 0.008; Fig. 11*A*). No significant group effect (*F*_(1,14)_ = 0.45, η_p_^2^ = 0.03, *p* = 0.514) or interaction (*F*_(1,14)_ = 2.10, η_p_^2^ = 0.13, *p* = 0.169) were found. Individual 98% confidence interval analysis of initial movement direction revealed significant interlimb transfer for 12/16 controls (11 leftward, 1 rightward) and no significant transfer for 5/16 controls, rightward transfer for patient MS (baseline average = 1.7 ± 2.3°, 98% CI [−0.7, 4.0], post 1 = 9.0°; Fig. 11*B*), leftward transfer for patient MM (baseline average = 0.2 ± 2.8°, 98% CI [−2.7, 3.1], post 1 = −6.8°; Fig. 11*C*) and leftward transfer for patient AM (baseline average = −3.7 ± 2.2°, 98% CI [−5.9, −1.4], post 1 = −10.2°; Fig. 11*D*). Individuals’ magnitude of transfer (post 1, baseline) can be seen in Figure 12*A*. According to Crawford’s modified *t* test, absolute interlimb transfer did not significantly differ between any of the patients and the control group (*n* = 16; controls = 4.9 ± 4.2°; MS = 7.3°, *z* = 0.57, *p* = 0.583; MM = 6.6°, *z* = 0.39, *p* = 0.698; AM = 7.0°, *z* = 0.49, *p* = 0.638; Fig. 11*B*). We also compared the magnitude of interlimb transfer of each patient to the controls who were classified as presenting interlimb transfer (*n* = 12). No significant difference was found in the absolute magnitude of transfer between these controls and patients using Crawford’s modified *t* test (controls = 6.2 ± 4.1°; MS = 7.3°, *z* = 0.26, *p* = 0.805; MM = 6.6°, *z* = −0.09, *p* = 0.932; AM = 7.0°, *z* = 0.190, *p* = 0.859). Finally, a 2 × 2 ANOVA (two groups, two arms) on the controls’ post 1 trials (absolute values) showed a significant effect of arm (*F*_(1,14)_ = 13.08, η_p_^2^ = 0.48, *p* = 0.003), with a significantly greater deviation of the DA than the NDA. There was no significant group or interaction effect. Correlation analysis performed between the control groups’ after-effect on the DA (−10.5 ± 5.3°) and transfer effect on the NDA (4.9 ± 4.2°) showed no significant correlation (*r* = −0.27, *p* = 0.922; for graphical presentation of the post values for controls and each patient, see Fig. 6). These results suggest that the magnitude of each individual’s after-effect and transfer effect were not related.

Results were further confirmed by analysis of maximum perpendicular deviation, as individual 98% confidence interval analysis showed significant interlimb transfer for the majority of controls, 13/16, and all three patients. Crawford’s modified *t* test showed no significant differences in the absolute magnitude of transfer between controls and patients (controls = 3.0 ± 1.8 cm; MS = 1.9 cm, *z* = −0.63, *p* = 0.549; MM = 2.8 cm, *z* = −0.12, *p* = 0.828; AM = 4.2 cm, *z* = 0.64, *p* = 0.539). Comparison of patients to controls classified as presenting interlimb transfer (*n* = 13) also showed no significant differences in the absolute magnitude of transfer using Crawford’s modified *t* test (controls = 3.5 ± 1.7 cm; MS = 1.9 cm, *z* = −0.95, *p* = 0.377; MM = 2.8 cm, *z* = −0.40, *p* = 0.706; AM = 4.2 cm, *z* = 0.42, *p* = 0.693). These results indicate that all three patients transferred the DA adaptation to the NDA despite their corpus callosum abnormalities.

## Discussion

We aimed to determine the role of the corpus callosum in interlimb transfer of sensorimotor adaptation in the context of unconstrained arm movements. Longstanding theoretical models of the neural mechanisms underlying interlimb transfer of motor learning highlighted the corpus callosum as a key structure mediating interhemispheric transfer of motor skills (Taylor and Heilman, 1980; Parlow and Kinsbourne, 1990). While certain studies have provided evidence toward these models (de Guise et al., 1999; Perez et al., 2007a; Bonzano et al., 2011), others have given evidence against (Thut et al., 1997; Criscimagna-Hemminger et al., 2003). Here, we found interlimb transfer of prism adaptation from the the dominant arm to the naïve non-dominant arm on an arm reaching task in three corpus callosum patients, with no significant difference in terms of magnitude compared with controls. The presence of interlimb transfer in each patient suggests that on an arm reaching task, interlimb transfer of prism adaptation does not require intact callosal pathways, notably those between bilateral motor, premotor, and supplementary motor areas. This would primarily suggest that the dominant theories of interlimb transfer involving the corpus callosum, developed mostly based on distal tasks, may not generalize to other tasks such as proximo-distal arm reaching. Further work is necessary to determine whether interlimb transfer relies on such pathways in healthy individuals. For instance, it is possible that the same neural mechanisms underly interlimb transfer in patients and healthy participants. On the other hand, the underlying mechanisms may differ, whereby the midbody of the corpus callosum may mediate interlimb transfer in healthy controls, whereas in the patients, brain plasticity mechanisms may have resulted in alternative neural mechanisms which maintain apparently normal interlimb transfer at the behavioral level.

### Comparable motor control and adaptation between corpus callosum patients and controls

Overall, in baseline reaching performance, patient MS, with recent stroke-induced lesions to the corpus callosum (preserving only the genu and splenium), and patient AM, with corpus callosum agenesis, showed few significant differences to controls. The only patient presenting substantial differences compared with controls was patient MM, who had recent stroke-induced lesions to the corpus callosum (preserving only the splenium). For instance, patient MM had no significant differences in initial movement direction and end point accuracy compared with controls, but showed abnormally slowed temporal kinematics, with a reduced peak velocity for both arms. Detrimental effects on temporal movement features such as slowing of unimanual arm reaching have been related to the degradation of corpus callosum pathways connecting premotor areas in stroke patients (Stewart et al., 2017). In addition, patient MM was tested only five months postinjury, which could have contributed to this motor slowing. Despite this, we did not find any significant difference between each patient and controls for spatial performance in baseline.

Each patient had a significant rightward prism effect and was able to reduce initial reaching errors caused by the initial perturbation. When examining early prism exposure, no significant difference between controls and the patients was found for the number of trials to reduce prism-induced errors, with fast error reduction based on visual feedback as in other reaching studies (Gréa et al., 2002; Pisella et al., 2004; Newport and Jackson, 2006; O’Shea et al., 2014; Renault et al., 2020). While awareness of the perturbation and strategic, possibly explicit, processes could partly underlie the rapid error reduction as well as adaptation and transfer, previous research (Mazzoni and Krakauer, 2006; Newport and Jackson, 2006; Taylor et al., 2011; Wang et al., 2011) suggests that this is unlikely to fully account for the present results. Finally, a significant leftward after-effect, often referred to as a hallmark of sensorimotor adaptation, was observed on the dominant arm (DA) across the control group and patients. The after-effect was equivalent to a deviation of −10.5 ± 5.3° for the control group and −11.7° to −15.9° for the two patients with a significant after-effect, MS and AM, respectively. The third patient, MM, had a non-significant after-effect of −1° at initial movement direction, but the after-effect was significant when looking at maximum perpendicular deviation. We did not find any significant correlation between the number of trials taken to de-adapt during the post phase and the magnitude of the after-effect, suggesting that the rate of NDA arm de-adaptation did not substantially affect the magnitude of the after-effect. The after-effect in our study (10.5°) corresponded to 61.4% of the prismatic deviation (17.1°), which was similar to the after-effect of 60.9% (9.1°) found by Facchin et al. (2019; their experiment 1) who used 15° right-ward deviating prisms over 150 adaptation trials, and, importantly, did not test opposite arm performance before after-effect assessment (see also Facchin et al., 2019; for a summary of the after-effects reported in the literature, see Table 1). Our findings thus support the idea of sensorimotor adaptation in each participant, offering the opportunity to assess interlimb transfer in patients and controls.

### Neural mechanisms of interlimb transfer

We hypothesized that corpus callosum abnormalities would interfere with interlimb transfer yet found interlimb transfer in each patient with either extensive midbody lesions or complete agenesis. Further, we found no significant difference in the magnitude of absolute interlimb transfer between each patient and matched controls. Across controls and patients, we did observe two profiles of interlimb transfer to the NDA arm: the majority with initial leftward deviation (opposite to the prismatic perturbation), consistent with encoding in extrinsic coordinates, and a few participants with initial rightward deviation (in the same direction as the prismatic perturbation), consistent with encoding in intrinsic coordinates. Overall, these findings support and extend those found on young, healthy individuals (Kalil and Freedman, 1966; Renault et al., 2020).

Regarding the underlying neural mechanisms of interlimb transfer, one could argue that the transfer observed in each patient could be because of the development of compensatory interhemispheric pathways through brain plasticity. Agenesis patients, like patient AM, often have preserved interhemispheric communication linked to the formation of alternative interhemispheric networks or upregulated information transfer via posterior or anterior commissures (Brescian et al., 2014; Tovar-Moll et al., 2014; Van Meer et al., 2016). In other pathologies such as split-brain patients, the presence and timeline of recovery of interhemispheric connectivity because of brain plasticity is less clear (for review, see Mancuso et al., 2019). In studies on split-brain patients, recovery of interhemispheric connectivity was shown two to seven years postsurgery in a group of patients (Roland et al., 2017) and decades postsurgery in two separate case studies (Uddin et al., 2008; Nomi et al., 2019). Here, we tested two stroke patients (MM and MS) within five months and two years postinjury, respectively. This short timescale reduces the likelihood of interhemispheric connectivity changes because of plasticity. Further, both patients had non-surgical, stroke-induced lesions following a normal development with no history of epilepsy, removing potential confounds of studying a surgically split brain because of severe epilepsy. While patient AM could have developed compensatory mechanisms for interlimb transfer during development, this explanation would be less likely for patient MS and patient MM.

One possibility is that preserved corpus callosum splenium fibers in patients MM and MS could underlie interlimb transfer. The splenium is known to connect bilateral posterior parietal, temporal, and visual areas (Zarei et al., 2006; Putnam et al., 2010), areas known to contribute to reach adaptation. In particular, posterior parietal areas underlie the planning and control of visually guided arm movements (Buneo and Andersen, 2006), while both posterior parietal and visual areas have been implicated in prismatic adaptation (Clower et al., 1996; Pisella et al., 2005; Luauté et al., 2009; Magnani et al., 2013; Crottaz-Herbette et al., 2014). While bilateral motor and premotor transcallosal connections were disrupted in patients MM and MS, it is possible that splenial connections could mediate transcallosal mechanisms of interlimb transfer between bilateral posterior parietal, temporal, or visual cortex areas. In line with this, in an agenesis patient like patient AM, visual areas normally connected via the splenium, were shown instead to be connected via the anterior commissure (Van Meer et al., 2016). Further work, for instance on other patients with rare stroke types affecting specifically the corpus callosum, and in particular the splenium, would thus be necessary to test this hypothesis.

An alternative hypothesis is that the observed interlimb transfer does not in fact rely on interhemispheric transfer and instead involves the dominant hemisphere (contralateral to the trained DA arm). Indeed, pioneering work on the neural basis of interlimb transfer (Taylor and Heilman, 1980) proposed that, for right-handed participants, the left hemisphere contains the effector-independent motor engram formed during learning. More recent research has further confirmed the implication of dominant left hemisphere networks in both motor control and adaptation with the right arm in right-handers (Dassonville et al., 1997; Buneo and Andersen, 2006; Luauté et al., 2009; Pool et al., 2014), including adaptation to rightward prisms (Panico et al., 2020; Schintu et al., 2020). Further, left hemisphere, but not right hemisphere, stroke patients show impaired adaptation to visuomotor rotations (Mutha et al., 2011). It is possible that the updated motor plans stored within the dominant hemisphere are accessible to the dominant limb but also the NDA limb, via ipsilateral cortico-spinal pathways rather than callosal pathways. Neurophysiological findings in healthy human and non-human primates have shown, for instance, that not only the contralateral hemisphere, but also the ipsilateral hemisphere can contribute to the execution of unimanual movements (Anguera et al., 2007; Luauté et al., 2009; Lee et al., 2010; Gabitov et al., 2016; Ames and Churchland, 2019; Heming et al., 2019). This is supported by clinical studies showing that unilateral stroke damage can affect the contralateral arm but also the ipsilateral arm (Desrosiers et al., 1996; Hermsdörfer et al., 1999; Schaefer et al., 2009), especially on proximal tasks (Jones et al., 1989). The role of ipsilateral descending pathways, comprising around 10–15% of all descending motor pathways to upper and lower arm extremities, is currently under intense investigation in both motor control and stroke rehabilitation research of the upper limb (Duque et al., 2008; Bradnam et al., 2013). Ipsilateral pathways appear to contribute more to proximal compared with distal effectors (Turton et al., 1996; Müller et al., 1997; Chen et al., 2003; Bawa et al., 2004), a finding which may be linked to reports that interlimb transfer is greater on proximal compared with distal tasks (Thut et al., 1997; Aune et al., 2017). Further, Criscimagna-Hemminger et al. (2003) showed interlimb transfer of force-field adaptation in a split-brain patient on a constrained proximo-distal reaching task, suggesting that such interlimb transfer does not rely on the corpus callosum and could be mediated by ipsilateral descending pathways. Studies finding interlimb transfer on distal (hand or finger) tasks, such as sequence learning or force tasks, however, implicate a key role of interhemispheric communication via the corpus callosum (Perez et al., 2007a; Lee et al., 2010; Bonzano et al., 2011; Ruddy and Carson, 2013; Gabitov et al., 2016). These results correspond to motor control observations in our patients, and other patients with corpus callosum abnormalities, showing that proximo-distal arm reaching performance can be largely unaffected while distal motor tasks are impaired (Gordon et al., 1971; Sauerwein and Lassonde, 1994). These findings, in combination with our results obtained on an unconstrained proximo-distal reaching task, could suggest that tasks involving distal effectors could require callosal pathways, while tasks involving proximal effectors could rely on ipsilateral descending pathways.

One final interpretation could be that subcortical structures such as the cerebellum could underlie this interlimb transfer. Day and Brown (2001) suggested that visuomotor integration of reaching movements involved subcortical regions, potentially the cerebellum, as an agenesis patient showed normal visuomotor reaching despite an absent corpus callosum and absent ipsilateral motor evoked responses to the lower arm muscles. Since, imaging studies have shown evidence for cerebellar recruitment in prismatic adaptation involving reaching movements (Luauté et al., 2009; Küper et al., 2014). Notably, rightward prismatic adaptation, shown to involve a dominantly left lateralized cortical network, also involves the subcortical contralateral right cerebellum (Panico et al., 2020; Schintu et al., 2020), reciprocally connected to left cortical areas including parietal and motor cortices (Kamali et al., 2010; Palesi et al., 2017). A wealth of cerebellar patient studies have also shown the role of the cerebellum in force-field and visuomotor adaptation (Smith and Shadmehr, 2005; Rabe et al., 2009; Donchin et al., 2012), and prism adaptation (Martin et al., 1996b; Pisella et al., 2005; Block and Bastian, 2012; Hanajima et al., 2015). However, while the cerebellum has been shown to play a role in adaptation, Block and Celnik (2013) showed that inhibitory cerebellar stimulation did not interfere with interlimb transfer, and only interfered with visuomotor adaptation. Contrarily, on a grasping task, Nowak et al. (2009) showed impaired interlimb transfer in cerebellar patients. As cerebellar contributions vary between different adaptation tasks (Rabe et al., 2009; Donchin et al., 2012), and given that different adaptation paradigms are not necessarily measuring the same process (Fleury et al., 2019), further work is necessary to determine whether interlimb transfer of prismatic adaptation is mediated by cerebellar mechanisms, involved in a parieto-cerebellar-motor network (Obayashi, 2004; Newport and Jackson, 2006).

In summary, our assessment of arm reaching performance in patients with corpus callosum abnormalities revealed interlimb transfer of prismatic adaptation, with no significant differences in the magnitude of transfer compared with matched controls. The presence of interlimb transfer in each patient suggests that on an arm reaching task, interlimb transfer of prism adaptation does not require intact callosal pathways, notably those between bilateral motor, premotor, and supplementary motor areas. This would primarily suggest that the dominant theories of interlimb transfer involving the corpus callosum, developed mostly based on distal tasks, may not generalize to other tasks such as proximo-distal arm reaching. Further work is necessary to determine whether interlimb transfer relies on such pathways in healthy individuals. For instance, it is possible that the same neural mechanisms underly interlimb transfer in patients and healthy participants. On the other hand, the underlying mechanisms may differ, whereby the midbody of the corpus callosum may mediate interlimb transfer in healthy controls, whereas in the patients, brain plasticity mechanisms may have resulted in alternative neural mechanisms which maintain an apparently normal profile of interlimb transfer at the behavioral level.

### Limitations

One possible limitation of the present study is that brain plasticity in corpus callosum patients could have resulted in alternate pathways for interlimb transfer of sensorimotor adaptation, which could otherwise rely on the midbody of the corpus callosum in a normal healthy brain. This limitation could be especially relevant for the agenesis patient as previous studies on agenesis subjects have shown upregulated functionality of the anterior commissure (Brescian et al., 2014; Tovar-Moll et al., 2014; Van Meer et al., 2016), ipsilateral descending pathways (Ziemann et al., 1999), and possibly subcortical pathways (Day and Brown, 2001). Further studies using functional brain imaging or brain stimulation would be necessary to give greater insights into the underlying neural mechanisms.

A second limitation is that we were able to work with a relatively small number of patients. This is because there is a low prevalence of agenesis and callosal lesions in stroke patients (Giroud and Dumas, 1995; Paul et al., 2007; Sun et al., 2019). For example stroke confined to the corpus callosum was observed in 21 of 5584 patients (0.4%) in the Shanghai study with a recruitment period of four years (Sun et al., 2019), and three of 282 patients (1%) in the French study with a recruitment period of one year (Giroud and Dumas, 1995). However, previous research has shown that even only one rare patient can be enough to reveal key insights in neuroscience, as evidenced by the Nobel-prize winning research on split-brain developed by Sperry and colleagues (Gazzaniga et al., 1962; Volz and Gazzaniga, 2017). Increasing sample size would not change our observations of interlimb transfer on all three patients, and thus our conclusion, that the midbody of the corpus callosum is not necessary for the interlimb transfer of prism adaptation. However, working with more patients, and especially patients with distinct lesions, would be helpful in clarifying the neural mechanisms underlying interlimb transfer. This is consistent with the idea that heterogenous samples can give greater neurologic insights (Martin et al., 1996a; Willems et al., 2014). Interestingly, while Sun et al. (2019) found high prevalence of splenium lesions, we, along with Giroud and Dumas (1995), found a preserved splenium in both stroke patients. A future study with patients presenting splenium lesions would be useful to test the hypothesis that interlimb transfer relies on interhemispheric transfer of information via the splenium.

Finally, the two stroke patients and the agenesis patient tested in our study were heterogenous in terms of laterality, sex and age, giving rise to a heterogenous control group. However, age characteristics did not appear to influence the results of visuomotor adaptation and interlimb transfer across participants. Further, on a similar prismatic adaptation study, no significant effect of laterality or sex was found in a larger group of control participants which was more homogenous in terms of age (Renault et al., 2020). Whilst we used adapted statistical analyses developed to estimate whether a single patient can be considered normal or abnormal compared with small or moderate control samples (Crawford and Garthwaite, 2007; Crawford et al., 2010), statistically non-significant results do not necessarily indicate complete lack of difference between patients and controls (Altman and Bland, 1995). Further studies with an increased number of control participants could be useful to clarify this.

## Synthesis

Reviewing Editor: Bradley Postle, University of Wisconsin Decisions are customarily a result of the Reviewing Editor and the peer reviewers coming together and discussing their recommendations until a consensus is reached. When revisions are invited, a fact-based synthesis statement explaining their decision and outlining what is needed to prepare a revision will be listed below. The following reviewer(s) agreed to reveal their identity: Mark Hinder.

R1

The manuscript "Interlimb transfer of reach adaptation does not require an intact corpus callosum: Evidence from patients with callosal lesions and agenesis", submitted to eNeuro (eN-NWR-0190-20), investigates the role of the Corpus Callosum interlimb transfer of sensorimotor adaptation.

The authors claim that sensorimotor adaptation can be

transfer across limbs despite extensive lesions or complete absence of the corpus callosum.

In my view, this could be a potentially interesting paper. However, there are some issues, which should be adequately addressed by the authors, as described below.

- Can the authors comment on the influence of the dominant hemisphere in the experiments and results?

-I do appreciate the diversity of the patients. However, to draw a conclusion, the study may have some limitations. For example: the subjects are heterogeneous and the sample size is too small. Could the results be different if they had a larger sample size and/or more homogenous? Please comment on this.

- The author affirms: "These results thus suggest that on an arm reaching task, interlimb transfer of prism adaptation does not require intact callosal pathways, notably those between bilateral motor, premotor and supplementary motor areas." - this statement might be differ depending on the contexts, such as for normal individuals and invidious with brain anomalies. Please also comment on the light of the concept of brain plasticity.

- Please comment on the limitation of the study, such as small sample size and no differences between patients and controls in a small and heterogeneous population. How the author can generalize this findings?

Figures:

It would be interesting to have a more detailed anatomical information about the subjects.

R2

Abstract is clear and well written

Introduction

The intro provides appropriate theoretical models, the cross activation model and the callosal access model. It might be prudent to also cite/describe Lee et al. (J Physiol 588.1 2010 pp 201-212), where rTMS was used to stimulate the trained or untrained hemisphere and suggested involvement of the ipsilateral M1 (i.e., the untrained M1).

Methods

I am unsure why there is such a long time between the tone to indicate participants could return to the target, and the end of the trial.

Did the return to start position occur with/without the start position being illuminated? For the return movement did participants also have to keep their finger above the table?

Line 194 - clarify here that these practice reaches were done without visuomotor rotation.

Lines 203 - why was dominant always done first ? Could this not have been counterbalanced/randomised ?

Some rationale for only looking at adaptation of the dominant arm is required, i.e., is transfer from training the dominant hand and looking for transfer in the non-dominant limb, as opposed to vice-versa?

Line 211 - why only train to one (middle target) ? please provide rationale

Line 217 - the post-phase looks at both transfer to the non-trained limb, but also to untrained targets. How can you assess the interlimb transfer to untrained targets when you don’t know the extent of the inter-target generalisation of learning in the trained limb? Please provide more rationale for which targets are trained on/transfer assessed on, as the description in 222-225 doesn’t seem related to the mechanism/degree of transfer, rather more to do with prediction of target direction (and I don’t follow how that relates to the current question of transfer).

Line 279 - rationale for including individual reaches as well as 10-reach averages within the same ANOVA requires some justification. If the intention was to look at rapid early adaptation why not look at trials 1-10 of adaptation separately, and then longer term adaptation by looking at 10-trial averages (1-10, 11-20, 21-30, etc). Also, if looking at dominant hand, the post1 trial occurs after the non-dominant limb has de-adapted. So this dominant post-1 trial might be affected by the rate of de-adaptation of the non-dominant limb. Does this need to be considered?

Results

Lines 352-54 does not read correctly, please check and revise

Figure 5 - why is 5D post1 trial (blue trace) shown as a dashed line while other panels have a solid line?

Line 374 and 375: p can’t be p = 0.0000, present as p<0.001 for example (also line 397)

Line 393 "P" not "p value"

Be consistent with the decimal places for all p value reporting

It would be useful to see the 30 post phase NDA trials, and then the post phase DA trials on a figure, together with the adaptation (prism) trials to see how the magnitude of the error in DA post trial 1 compares to NDA post trial 1. Is this comparison undertaken directly? This does seem to be a missing aspect of the results.

Overall the individual comparisons of each patient to the control group/s via the modified t-test analyses is a nice way to show the patients individualised characteristics.

Discussion

Given the lack of neurophysiological data (TMS, for example), the mechanisms that underpin the preserved transfer and aftereffects is quite speculative, though quite well presented and theoretically feasible and consistent with current understanding and previous results.
